# Supplementary material for: Potent transmission-blocking monoclonal antibodies from naturally exposed individuals target a conserved epitope on Plasmodium falciparum Pfs230
Source: Immunity. 2023 Feb 14;56(2):420–432.e7. doi: 10.1016/j.immuni.2023.01.013 (PMC9942874; doi:10.1016/j.immuni.2023.01.013)
Supplement: Document S1. Figures S1–S6 and Tables S3, S4, and S6 [file mmc1.pdf]

Immunity, Volume *56*

Supplemental information

Potent transmission-blocking monoclonal antibodies from naturally exposed individuals target a conserved epitope on *Plasmodium falciparum* Pfs230

Danton Ivanochko, Amanda Fabra-García, Karina Teelen, Marga van de Vegte-Bolmer, Geert-Jan van Gemert, Jocelyn Newton, Anthony Semesi, Marloes de Bruijni, Judith Bolscher, Jordache Ramjith, Marta Szabat, Stefanie Vogt, Lucas Kraft, Sherie Duncan, Shwu-Maan Lee, Moses R. Kamya, Margaret E. Feeney, Prasanna Jagannathan, Bryan Greenhouse, Robert W. Sauerwein, C. Richter King, Randall S. MacGill, Teun Bousema, Matthijs M. Jore, and Jean-Philippe Julien

Supplementary Figures and Tables

Potent transmission-blocking monoclonal antibodies from naturally exposed individuals target a conserved epitope on *Plasmodium falciparum* Pfs230

Danton Ivanochko[1*], Amanda Fabra-García[2*], Karina Teelen[2], Marga van de Vegte-Bolmer[2], Geert-Jan van Gemert[2], Jocelyn Newton[1], Anthony Semesi[1], Marloes de Bruijni[3], Judith Bolscher[3], Jordache Ramjith[4], Marta Szabat[5], Stefanie Vogt[5], Lucas Kraft[5], Sherie Duncan[5], Shwu-Maan Lee[6], Moses R Kamya[7], Margaret E Feeney[8,9], Prasanna Jagannathan[10], Bryan Greenhouse[8], Robert W Sauerwein[3], C Richter King[6], Randall S MacGill[6], Teun Bousema[2,#], Matthijs M Jore[2,#], Jean-Philippe Julien[1,11,#]

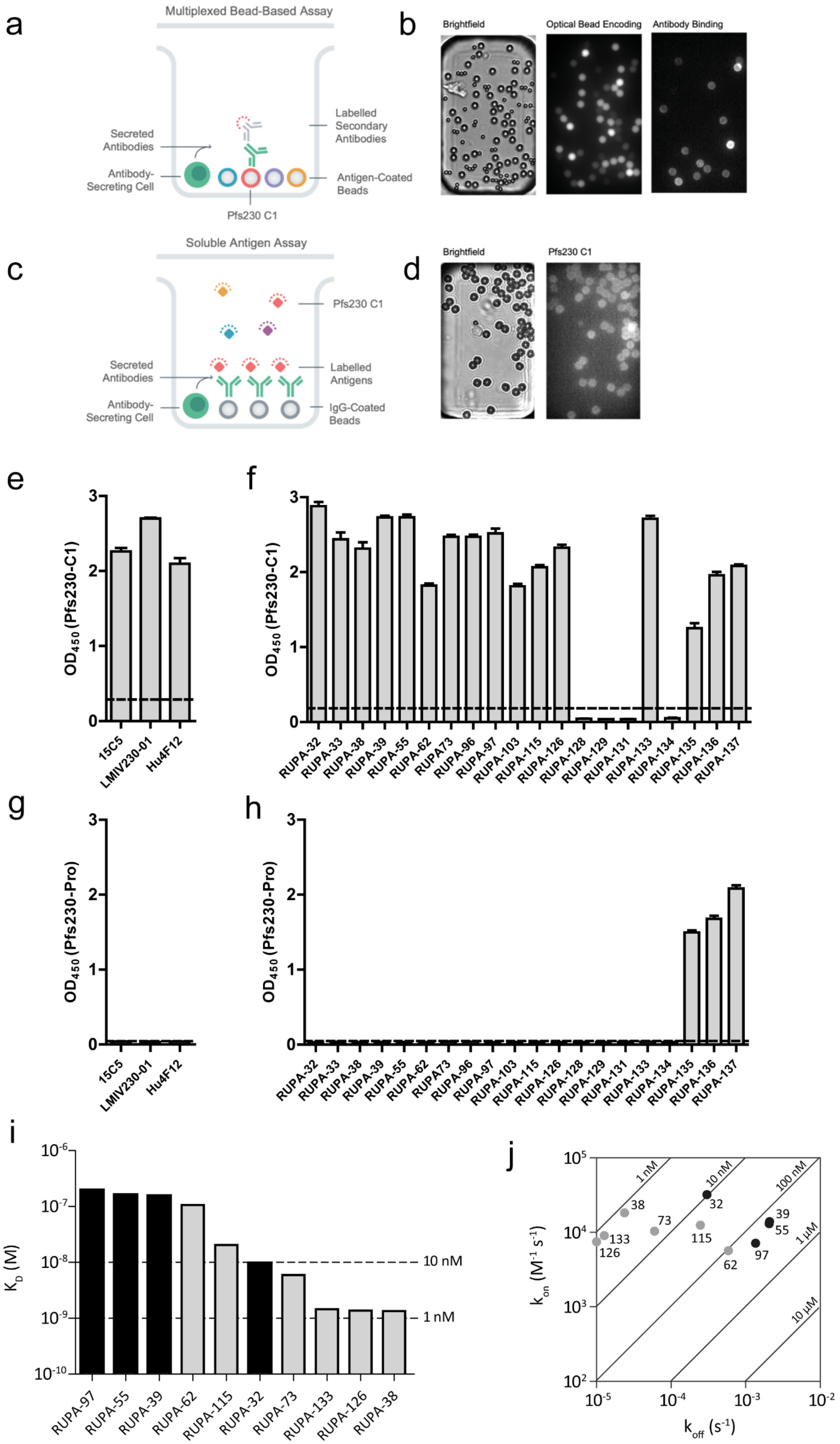
a
Multiplexed Bead-Based Assay
Secreted Antibodies
Antibody-Secreting Cell
Labelled Secondary Antibodies
Antigen-Coated Beads
Pfs230 C1
b
Brightfield
Optical Bead Encoding
Antibody Binding
c
Soluble Antigen Assay
Pfs230 C1
Secreted Antibodies
Labelled Antigens
Antibody-Secreting Cell
IgG-Coated Beads
d
Brightfield
Pfs230 C1
e
OD450 (Pfs230-C1)
15C5
LMIV230-01
Hu4F12
f
OD450 (Pfs230-C1)
RUPA-32
RUPA-33
RUPA-38
RUPA-39
RUPA-55
RUPA-62
RUPA73
RUPA-96
RUPA-97
RUPA-103
RUPA-115
RUPA-126
RUPA-128
RUPA-129
RUPA-131
RUPA-133
RUPA-134
RUPA-135
RUPA-136
RUPA-137
g
OD450 (Pfs230-Pro)
h
OD450 (Pfs230-Pro)
i
KD (M)
10 nM
1 nM
RUPA-97
RUPA-55
RUPA-39
RUPA-62
RUPA-115
RUPA-32
RUPA-73
RUPA-133
RUPA-126
RUPA-38
j
kon (M-1 s-1)
koff (s-1)
1 nM
10 nM
100 nM
1 μM
10 μM

Fig S1. Single B cell screening assays, domain-specificity of identified mAbs, and affinities of mAbs for Pfs230-C1. Related to **Fig 1** and **Fig 2**. (**a, c**) Representations of the multiplexed bead-based and soluble antigen assays using during primary screening. (**b, d**) Representative microscopic screening images of antibodies assessed for specificity to Pfs230 C1 in each assay. Enzyme-linked immunosorbent assays were performed with Pf230-C1 and Pfs230-Pro constructs to determine domain specificity. Previously identified mAbs against Domain I recognized Pfs230-C1 (**e**), but not Pfs230-Pro (**g**). 16 of the 20 identified mAbs bound to Pfs230-C1 (**f**). Three of these antibodies also react with Pfs230-Pro (**h**). All mAbs were tested at 10 μg/mL. Values are means of three technical replicates and error bars show s.e.m. Dashed lines represent the mean signal + three standard deviations of eight non-Pfs230 mAbs and were used as cutoff to determine positivity. (**i**) Affinity and (**j**) binding kinetics were determined using surface plasmon resonance with immobilized antibodies and antigen as analyte. Black bars and dots represent mAbs with high transmission reducing activity. K_D, binding affinity; k_{on}, association constant; k_{off}, dissociation constant.

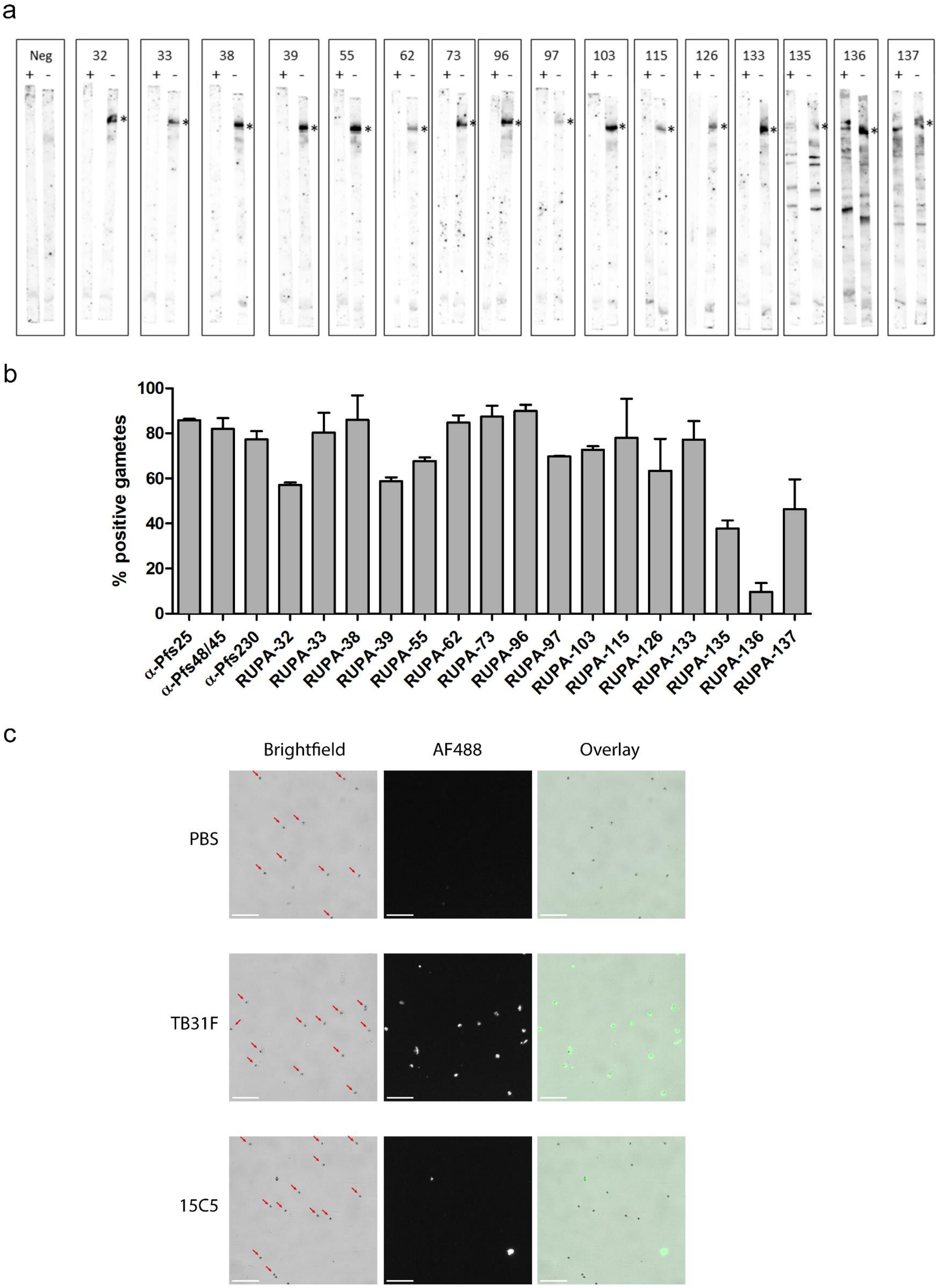

a
Neg
32
33
38
39
55
62
73
96
97
103
115
126
133
135
136
137
+ -
*
b
% positive gametes
100
80
60
40
20
0
α-Pfs25
α-Pfs48/45
α-Pfs230
RUPA-32
RUPA-33
RUPA-38
RUPA-39
RUPA-55
RUPA-62
RUPA-73
RUPA-96
RUPA-97
RUPA-103
RUPA-115
RUPA-126
RUPA-133
RUPA-135
RUPA-136
RUPA-137
c
Brightfield
AF488
Overlay
PBS
TB31F
15C5

Fig S2. Antibody recognition of native Pfs230 by Western blot and surface immunofluorescence assay. Related to **Fig 1**. (**a**) Gametocyte extract was separated on an SDS-PAGE gel under reducing (+) and non-reducing (-) conditions and transferred to a nitrocellulose membrane. Membrane strips were then incubated with different mAbs. Pfs230 band is indicated by asterisks. Neg: non-targeting mAb. (**b**) For SIFAs, all antibodies were tested at 5 µg/mL, except for mAbs against Pfs25 (2544, 1 µg/mL), Pfs48/45 (TB31F, 0.1 µg/mL) and Pfs230 (Hu2A2, 1 µg/mL). Anti-PfCSP mAb 399, which is sporozoite specific and does not recognize female gametes, was used for background correction. Values represent means of two technical replicates after background correction, and error bars show s.d. (**c**) For the micrograph showing that 15C5 does not react with female gametes in surface immunofluorescence assay, TB31F and PBS were included as a positive and negative control respectively. Both TB31F and 15C5 were tested at 3.2 µg/mL. Bound human antibody was detected with anti-human IgG-AF488. Gametes in brightfield channel are indicated with red arrows. Scale bar represents 50 micrometers.

Table S3. X-ray crystallography data collection and refinement statistics.
(Related to **Fig 3** and **Fig 4**.

	Pfs230D1+ and Fab RUPA-32	Pfs230D1+ and Fab RUPA-38	Pfs230D1+ and Fab RUPA-55	Pfs230D1+, Fab RUPA-97, and Fab 15C5	Pfs230D1+ and Fab LMIV230-02
**PDB ID**	7UVH	7UVO	7UVI	7UVQ	7UVS
**Beamline**	APS 23-ID-D	APS 23-ID-B	APS 23-ID-B	APS 23-ID-D	APS 23-ID-B
**Wavelength (Å)**	1.033190	1.033192	1.033167	1.033190	1.033167
**Space group**	C2	C2	I222	$P6_2$	C2
**Cell dimensions**					
**a,b,c (Å)**	154.87, 80.47, 124.96	163.71, 42.85, 95.30	91.02, 100.78, 425.75	155.00, 155.00, 92.90	92.36, 132.47, 125.07
**α, β, γ (º)**	90.0, 111.8, 90.0	90.0, 102.4, 90.0	90.0, 90.0,90.0	90.0, 90.0,120.0	90.0, 102.3, 90.0
**Resolution (Å)**	29.43 - 2.59 (2.68 - 2.59)	29.06 – 2.09 (2.16 – 2.09)	29.67 – 2.92 (3.02 – 2.92)	29.76 – 3.29 (3.41 – 3.29)	29.85 – 2.03 (2.10 – 2.03)
**Total reflections**	540,791 (54,145)	465,926 (34,888)	1,157,261 (118,833)	324,685 (32,881)	651,789 (52,806)
**Unique reflections**	44,578 (4413)	38,739 (3849)	43,033 (4247)	19,432 (1925)	93,278 (8599)
**Multiplicity**	12.1 (12.3)	12.0 (9.1)	26.9 (28.0)	16.7 (17.1)	7.0 (6.1)
**R_{meas}**	0.222 (2.156)	0.219 (1.630)	0.277 (3.627)	0.150 (2.009)	0.124 (1.160)
**R_{pim}**	0.064 (0.615)	0.062 (0.526)	0.054 (0.679)	0.036 (0.481)	0.046 (0.458)
**$\langle I/\sigma\ I \rangle$**	9.5 (1.6)	7.7 (1.6)	11.5 (1.7)	14.7 (1.7)	11.1 (1.6)
**$CC_{1/2}$**	0.995 (0.696)	0.995 (0.622)	0.998 (0.786)	0.999 (0.648)	0.998 (0.667)
**Completeness (%)**	100 (100)	99.8 (99.4)	99.8 (99.8)	100 (100)	98.6 (90.6)
**Refinement Statistics**					
**R_{work}/R_{free} (%)**	19.6/23.3	21.7/23.5	21.8/23.7	21.5/26.3	17.4/21.5
**Reflections for R_{free}**	1999	1937	2233	987	2019
**Molecules in ASU**	2	1	2	1	2
**Non-H atoms**	9382	4861	9476	8062	10,235
**Macromolecule**	9254	4743	9476	8062	9521
**Water**	88	142	0	0	713
**Heteroatom**	40	6	0	0	1
**RMSD bonds (Å)**	0.011	0.0068	0.0042	0.014	0.0079
**RMSD angles (°)**	1.39	1.01	0.94	1.64	1.05
**Ramachandran statistics**					
**Favored (%)**	97.9	95.9	95.8	96.4	97.7
**Allowed (%)**	2.1	4.1	4.2	3.6	2.3
**Outliers (%)**	0	0	0	0	0
**B-factors ($Å^2$)**					
**Wilson B-value**	57.4	36.7	75.7	121.8	31.6
**Average B-factors**	77.6	64.1	106.5	150.7	49.3
**Average macromolecule**	77.7	64.9	106.5	150.7	49.6
**Average water molecule**	62.0	40.3	-	-	45.5
**Average heteroatom**	80.8	29.1	-	-	52.2

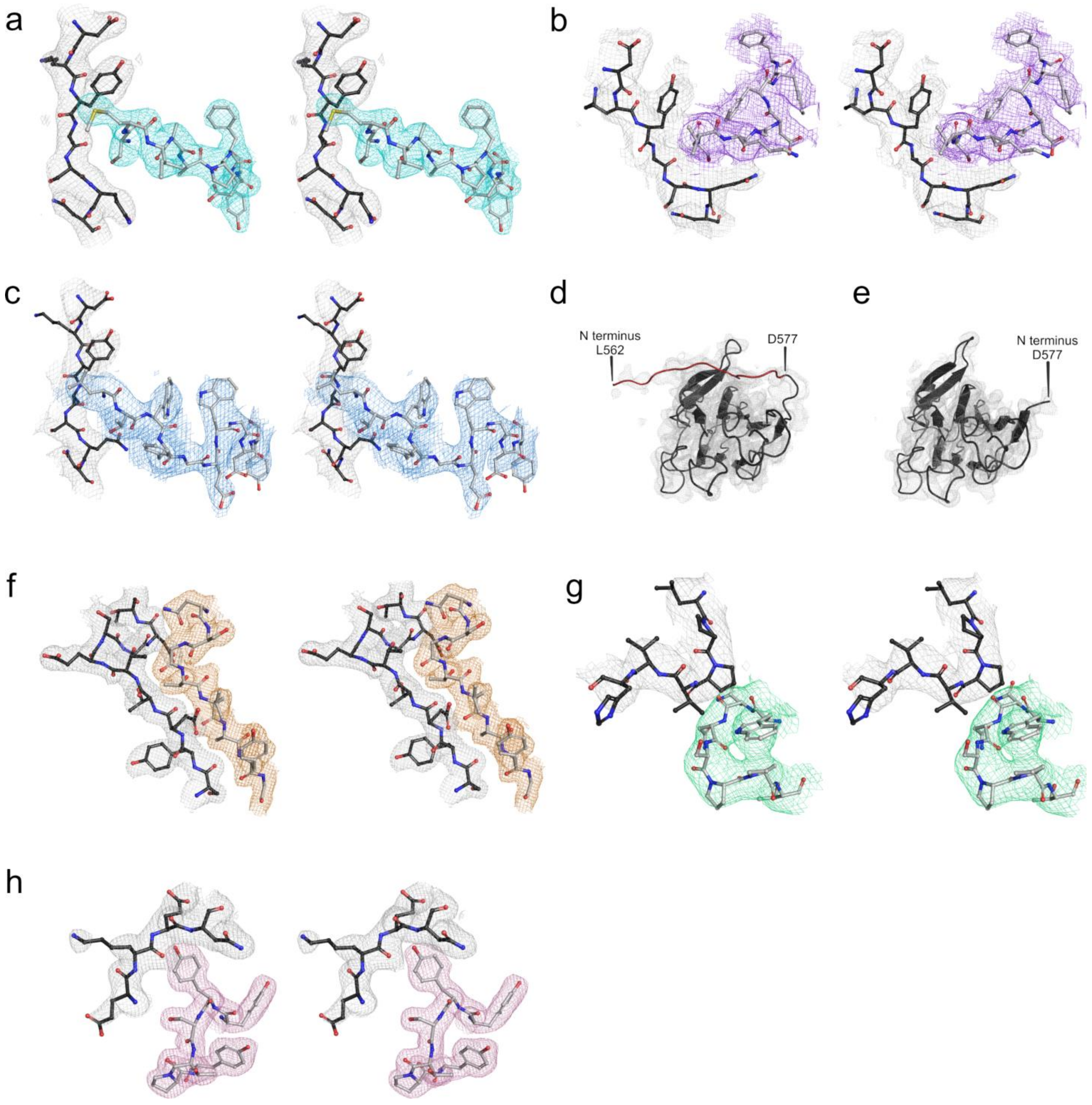

Fig S3. Experimental details of Fab-antigen interactions and Pfs230 D1 structures. Related to **Fig 3** and **Fig 4**. Stereo images of representative composite omit maps depicting electron density contoured at 1.0 sigma around Pfs230 D1+ bound by high potency Fabs (**a**) RUPA -32, (**b**) RUPA-55, and (**c**) RUPA-97, coloured in cyan, purple, and blue, respectively. Composite omit maps depicting electron density contoured at 1.0 sigma around the two biological assemblies of Pfs230 D1+ from the Fab RUPA-55 bound structure observed in (**d**) chain C and (**e**) chain F. The unique residue sequence (amino acids 562 to 576) is highlighted in red. Stereo images of representative composite omit maps depicting electron density contoured at 1.0 sigma around Pfs230 D1+ bound by low potency Fabs, (**f**) RUPA-38, (**g**) 15C5, and (**h**) LMIV230-02, coloured in orange, green, and pink, respectively.

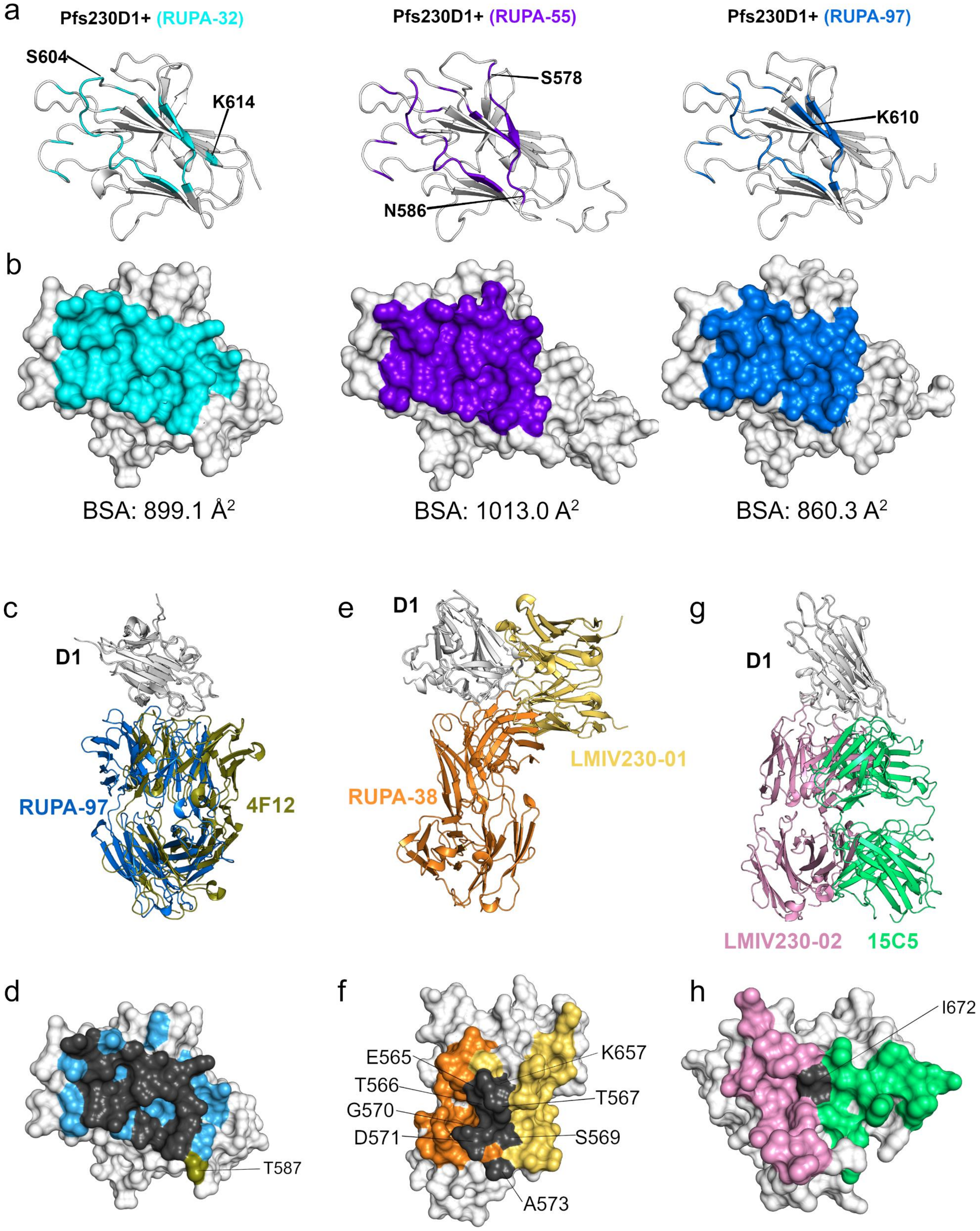
a
Pfs230D1+ (RUPA-32)
S604
K614
Pfs230D1+ (RUPA-55)
S578
N586
Pfs230D1+ (RUPA-97)
K610
b
BSA: 899.1 Å²
BSA: 1013.0 A²
BSA: 860.3 A²
c
D1
RUPA-97
4F12
e
D1
LMIV230-01
RUPA-38
g
D1
LMIV230-02
15C5
d
T587
f
E565
K657
T566
T567
G570
D571
S569
A573
h
I672

Fig S4. Epitopes of high potency transmission-reducing mAbs and other overlapping epitopes on Pfs230 D1. Related to **Fig 3**. The epitopes on Pfs230 D1+ of RUPA-32, -55, and -97 are coloured in cyan, purple, and blue, respectively, shown as (**a**) cartoon and (**b**) surface representations. Unique residues within each epitope are labeled. (**c**) Structural superposition on Pfs230 domain 1 of the Fab RUPA-97 structure with the structure of 4F12 (PDB ID 6OHG). (**d**) Surface representation of Pfs230 D1+ showing the epitopes of RUPA-32, -55, and -97 (light blue) and 4F12 (olive). (**e**) Structural superposition on the Pfs230 domain 1 of the RUPA-38 structure with the structure of scFv LMIV230-01 (PDB ID 7JUM). (**f**) Surface representation of Pfs230 D1+ showing the epitopes of RUPA-38 (orange) and LMIV230-01 (yellow). (**g**) Structural superposition on Pfs230 domain 1 of the Fab LMIV230-02 structure with the structure of Fab 15C5. (**h**) Surface representation of Pfs230 D1+ showing the epitopes of LMIV230-02 (pink) and 15C5 (green). All the overlapping epitopes are coloured in dark gray, and residues are labeled.

Table S4. Co-occurring SNPs present in Pfs230 D1+ amino acids 552 - 731. Related to **Table 1**. No SNPs within epitope bin I (A583T, T602K, E612K, V632A, K716N, and N719S) were found to co-occur with each other, while E612K, A583, and K716 (indicated in bold) co-occurred with SNPs found on other regions of D1+. Since G605S occurs with an allele frequency of 94.4%, the 5.6% of G605 alleles are reported herein.

Co-occurring SNPs	Freq (%)
K661N + D713Y	1.912
T652R + K661N	1.814
G605 + K661N	0.998
T656N + K661N	0.689
E655V + K661N	0.323
K661N + A699T	0.056
K661N + V687I	0.056
G605 + D713Y	0.042
A699T + D713Y	0.014
D561N + K661N	0.014
D637N + K661N	0.014
**E612K** + K661N	0.014
E654K + K661N	0.014
G605 + N715K	0.014
K644Q + K661N	0.014
K661N + H697Q	0.014
K661N + N715K	0.014
K661N + T675K	0.014
K661N + V701M	0.014
K661N + Y726H	0.014
T652R + D713Y	0.014
T652R + K665Q	0.014
V574I + K661N	0.014
T652R + K661N + D713Y	0.351
G605 + T652R + K661N	0.267
E655V + K661N + D713Y	0.070
T652R + T656N + K661N	0.070
K661N + A699T + D713Y	0.042
T652R + E655V + K661N	0.028
T652R + K661N + A699T	0.028
**A583T** + G605 + K661N	0.014
E655V + K661N + A699T	0.014
G605 + K661N + A699T	0.014
G605 + K661N + D713Y	0.014
K661N + D713Y + **K716N**	0.014
K661N + T675K + D713Y	0.014
T656N + K661N + A699T	0.014
T656N + K661N + D713Y	0.014
T652R + K661T + K661N + D713Y	0.042
G605 + E655V + K661N + D713Y	0.014
G605 + T652R + K661N + D713Y	0.014
G605 + T652R + T656N + K661N	0.014
T652R + T656N + K661N + D713Y	0.014
T656N + K661N + A699T + D713Y	0.014

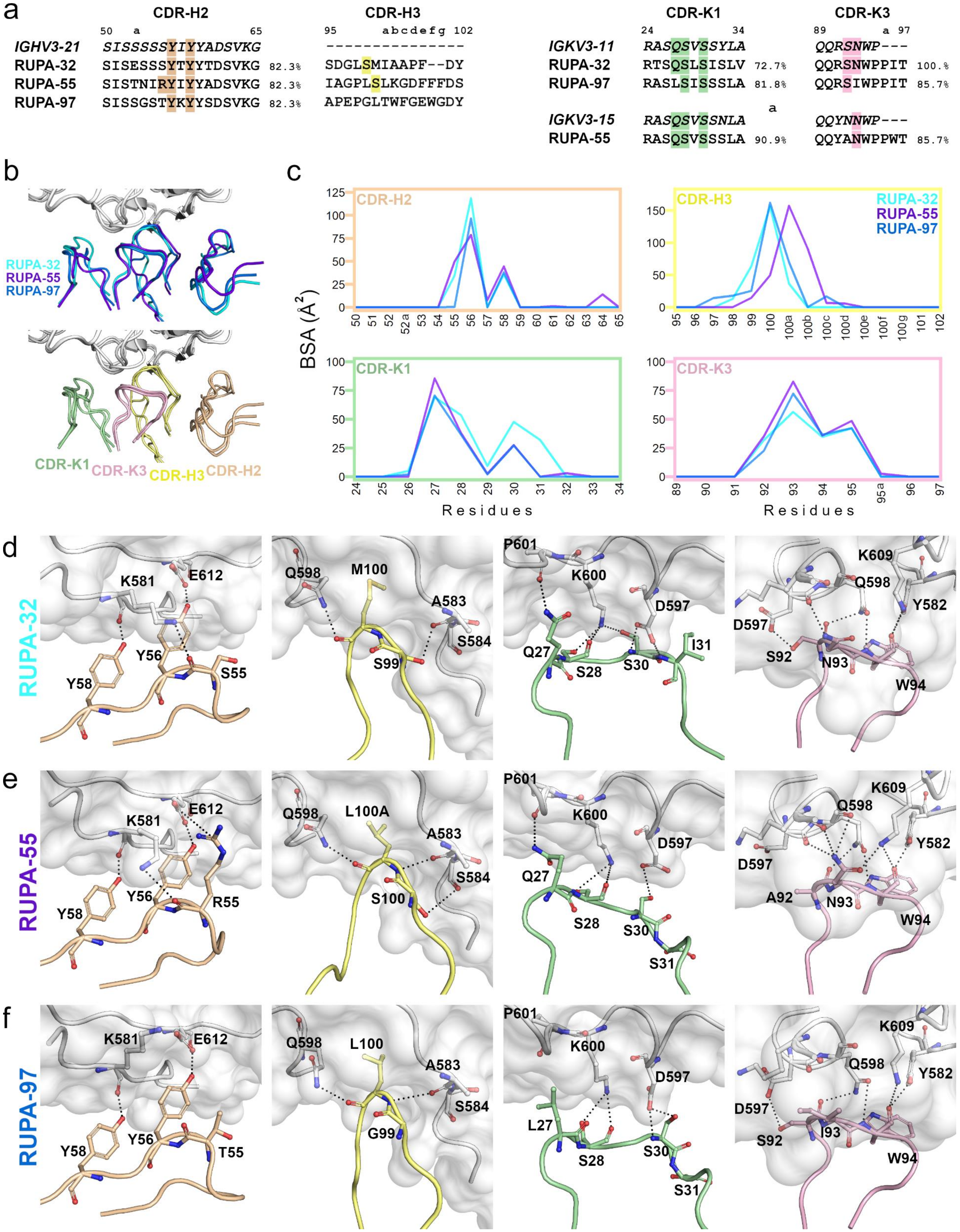
a
CDR-H2
CDR-H3
CDR-K1
CDR-K3
50 a 65
95 abcdefg 102
24 34
89 a 97
IGHV3-21 SISSSSSYIYYADSVKG
RUPA-32 SISESSSYTYYTDSVKG 82.3%
RUPA-55 SISTNIRYIYYADSVKG 82.3%
RUPA-97 SISSGSTYKYYSDSVKG 82.3%

SDGLSMIAAPF--DY
IAGPLSLKGDFFFDS
APEPGLTWFGEWGDY
IGKV3-11 RASQSVSSYLA QQRSNWP---
RUPA-32 RTSQSLSISLV 72.7% QQRSNWPPIT 100.%
RUPA-97 RASLSISSSLA 81.8% QQRSIWPPIT 85.7%
a
IGKV3-15 RASQSVSSNLA QQYNNWP---
RUPA-55 RASQSVSSSLA 90.9% QQYANWPPWT 85.7%
b
RUPA-32
RUPA-55
RUPA-97
CDR-K1
CDR-K3
CDR-H3
CDR-H2
c
BSA (Å²)
Residues
CDR-H2
CDR-H3
CDR-K1
CDR-K3
RUPA-32
RUPA-55
RUPA-97
d
RUPA-32
K581
E612
Y56
S55
Y58
Q598
M100
A583
S584
S99
P601
K600
D597
Q27
S28
S30
I31
K609
Q598
Y582
D597
S92
N93
W94
e
RUPA-55
K581
E612
Y56
R55
Y58
Q598
L100A
A583
S584
S100
P601
K600
D597
Q27
S28
S30
S31
K609
Q598
Y582
D597
A92
N93
W94
f
RUPA-97
K581
E612
Y56
T55
Y58
Q598
L100
A583
S584
G99
P601
K600
D597
L27
S28
S30
S31
K609
Q598
Y582
D597
S92
I93
W94

Fig S5. Detailed interactions of high potency Pfs230 D1+ binding mAbs. Related to **Table S3** and **Fig 3**. (**a**) Amino acid sequence alignments of antigen-interacting CDRs with the predicted germline precursor sequences. Residues forming H-bonds are highlighted in color. Kabat numbering is used. (**b**) Superposition of all antigen-interacting CDRs of RUPA-32, 55, and 97 in complex with Pfs230 D1+. (**c**) Plots of buried surface area (BSA) contributions by CDR residue. (**d-f**) Detailed interaction interfaces of CDRs K1, K3, H3, and H2 (coloured green, pink, yellow, and tan, respectively) for RUPA-32, 55, and 97. H-bonding interactions are indicated with dashed lines.

Table S6. Hydrogen-bonding interactions between high TRA mAbs and Pfs230 D1+. Related to **Fig 3**. The asterisk (*) denotes a salt bridge.

**Pfs230 D1+**	**RUPA-32**	**RUPA-55**	**RUPA-97**
K581	S55	R55	
K581	Y58	Y58	Y58
Y582	W94	W94	W94
A583	S99	L100a	L100
S584		S100	
D597	S30	S30	S30
D597	I31		
D597	S92		S92
D597	N93	N93	N93
Q598	S92	A92	S92
Q598		N93	
Q598	W94	W94	W94
Q598	M100		L100
Q598		L100a	
K600	S28	S28	S28
K600	S30		
K600		N93	
P601	Q27	Q27	
T602		E1	
E603		E1	
K609		N93	
K609	W94	W94	W94
E612*		R55*	
E612	Y56	Y56	Y56

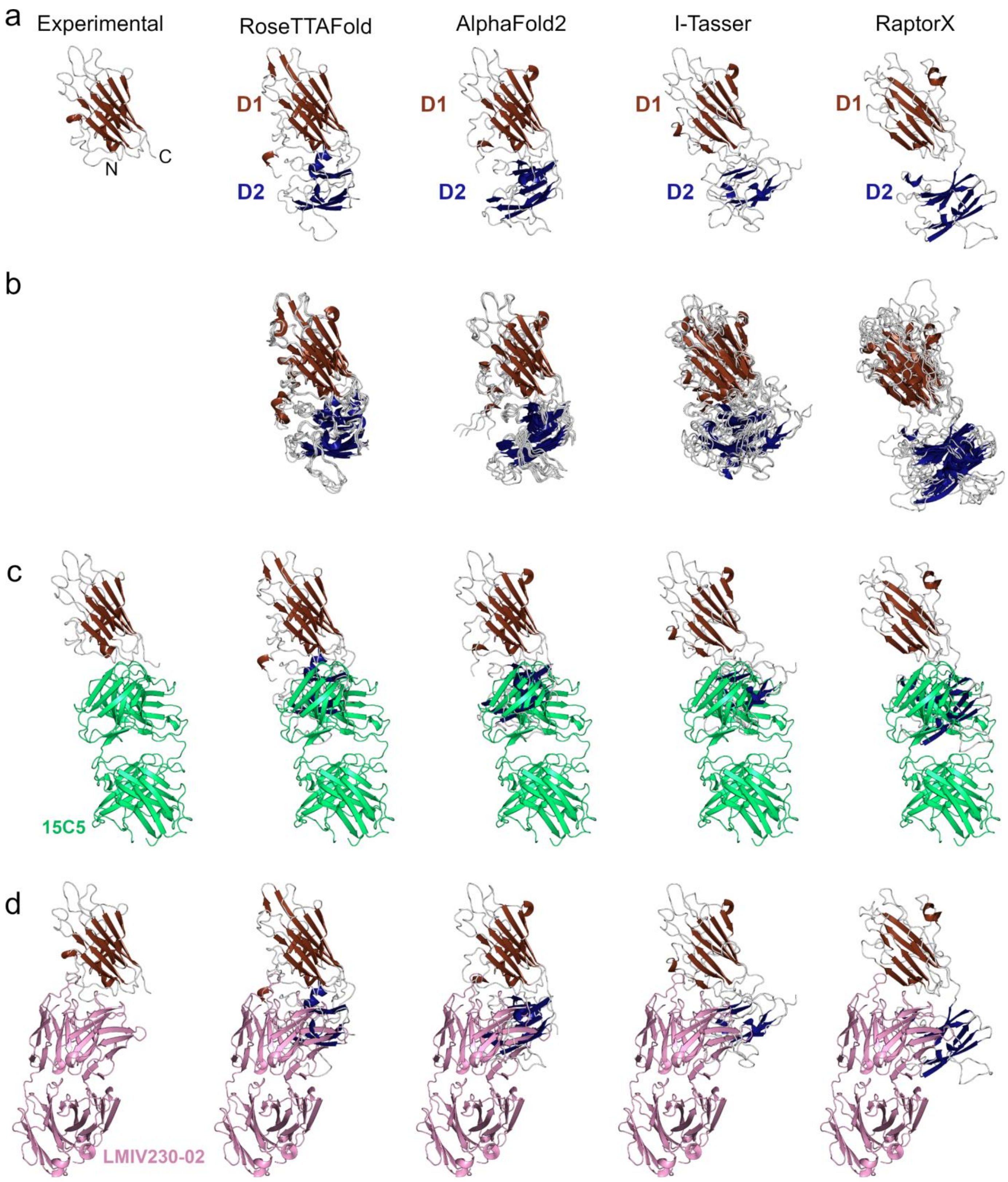

a
Experimental
RoseTTAFold
AlphaFold2
I-Tasser
RaptorX
N
C
D1
D2
D1
D2
D1
D2
D1
D2
b
c
15C5
d
LMIV230-02

Fig S6. Pfs230 domain 2 sterically occludes low-potency epitopes on domain 1. Related to **Fig 4**. (**a**) The crystal structure of Pfs230 D1+ (from the LMIV230-02 complex) contrasted against highest ranked theoretical models of domains 1 and 2 produced by RoseTTAFold[26], AlphaFold2, I-Tasser[27], and RaptorX[28]. (**b**) Superposition at domain 1 of all five output models from each *in silico* modeling approach demonstrates a general convergence for the position of domain 2. Superposition of Fabs (**c**) 15C5 and (**d**) LMIV230-02 onto their respective domain 1 epitopes on the theoretical models of domains 1 and 2 show clashing, independent of which modeling software is used.
