# Supplementary material for: Potent transmission-blocking monoclonal antibodies from naturally exposed individuals target a conserved epitope on Plasmodium falciparum Pfs230
Source: Immunity. 2023 Feb 14;56(2):420–432.e7. doi: 10.1016/j.immuni.2023.01.013 (PMC9942874; doi:10.1016/j.immuni.2023.01.013)
Supplement: Document S2. Article plus supplemental information [file mmc5.pdf]

Immunity

Article

Potent transmission-blocking monoclonal antibodies from naturally exposed individuals target a conserved epitope on *Plasmodium falciparum* Pfs230

Graphical abstract

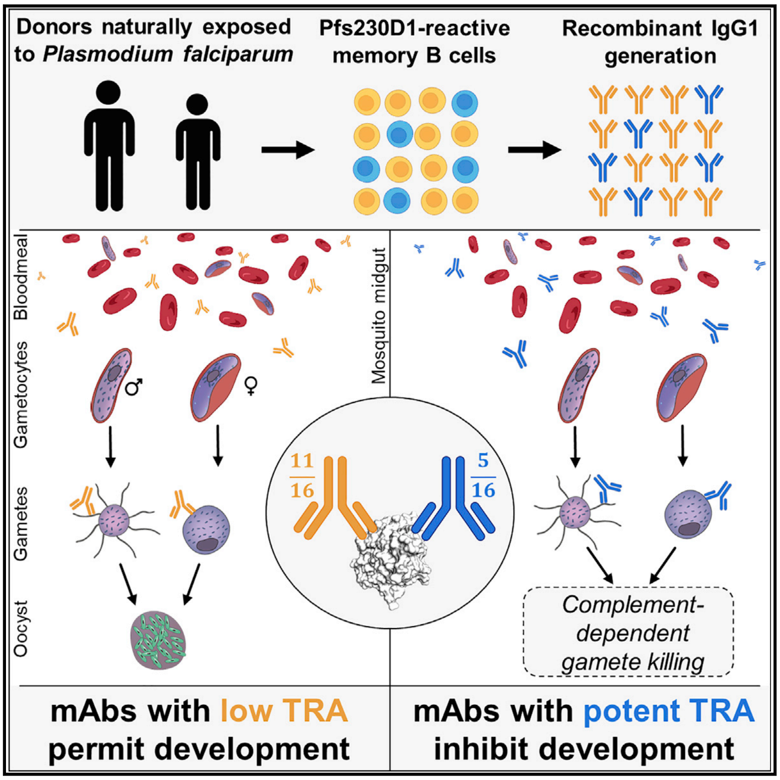

Highlights

- Isolated mAbs directed to Pfs230 elicited in naturally exposed individuals
- Highly potent transmission-reducing mAbs identified that target Pfs230 domain 1
- Structural delineation reveals potent and non-functional epitopes on Pfs230 domain 1

Authors

Danton Ivanochko, Amanda Fabra-García, Karina Teelen, ..., Teun Bousema, Matthijs M. Jore, Jean-Philippe Julien

Correspondence

teun.bousema@radboudumc.nl (T.B.), matthijs.jore@radboudumc.nl (M.M.J.), jean-philippe.julien@sickkids.ca (J.-P.J.)

In brief

Pfs230 domain 1 represents the most clinically advanced transmission-blocking vaccine candidate, but how human antibodies acquired after natural exposure recognize the protein is unknown. Ivanochko et al. report a panel of human mAbs targeting Pfs230 D1, including the most potent mAbs reported to date, and contrast structure-activity relationships against this malarial antigen to reveal the determinants of transmission-reducing activity.

Ivanochko et al., 2023, Immunity *56*, 420–432
February 14, 2023 © 2023 The Authors. Published by Elsevier Inc.
https://doi.org/10.1016/j.immuni.2023.01.013

Immunity

Article

Potent transmission-blocking monoclonal antibodies from naturally exposed individuals target a conserved epitope on *Plasmodium falciparum* Pfs230

Danton Ivanochko,[1,12] Amanda Fabra-García,[2,12] Karina Teelen,[2] Marga van de Vegte-Bolmer,[2] Geert-Jan van Gemert,[2] Jocelyn Newton,[1] Anthony Semesi,[1] Marloes de Bruijni,[3] Judith Bolscher,[3] Jordache Ramjith,[4] Marta Szabat,[5] Stefanie Vogt,[5] Lucas Kraft,[5] Sherie Duncan,[5] Shwu-Maan Lee,[6] Moses R. Kamya,[7] Margaret E. Feeney,[8,9] Prasanna Jagannathan,[10] Bryan Greenhouse,[8] Robert W. Sauerwein,[3] C. Richter King,[6] Randall S. MacGill,[6] Teun Bousema,[2,*] Matthijs M. Jore,[2,*] and Jean-Philippe Julien[1,11,13,*]

[1]Program in Molecular Medicine, the Hospital for Sick Children Research Institute, Toronto, ON, Canada
[2]Department of Medical Microbiology, Radboudumc, Nijmegen, the Netherlands
[3]TropIQ Health Sciences, Nijmegen, the Netherlands
[4]Radboud Institute for Health Sciences, Department for Health Evidence, Biostatistics Section, Radboudumc, Nijmegen, the Netherlands
[5]AbCellera Biologics Inc., Vancouver, BC, Canada
[6]PATH's Malaria Vaccine Initiative, Washington, DC 20001, USA
[7]Infectious Disease Research Collaboration, Kampala, Uganda
[8]Department of Medicine, University of California, San Francisco, San Francisco, CA, USA
[9]Department of Pediatrics, University of California, San Francisco, San Francisco, CA, USA
[10]Department of Microbiology and Immunology, Stanford University, Stanford, CA, USA
[11]Departments of Biochemistry and Immunology, University of Toronto, Toronto, ON, Canada
[12]These authors contributed equally
[13]Lead contact
*Correspondence: teun.bousema@radboudumc.nl (T.B.), matthijs.jore@radboudumc.nl (M.M.J.), jean-philippe.julien@sickkids.ca (J.-P.J.)
https://doi.org/10.1016/j.immuni.2023.01.013

SUMMARY

Pfs230 is essential for *Plasmodium falciparum* transmission to mosquitoes and is the protein targeted by the most advanced malaria-transmission-blocking vaccine candidate. Prior understanding of functional epitopes on Pfs230 is based on two monoclonal antibodies (mAbs) with moderate transmission-reducing activity (TRA), elicited from subunit immunization. Here, we screened the B cell repertoire of two naturally exposed individuals possessing serum TRA and identified five potent mAbs from sixteen Pfs230 domain-1-specific mAbs. Structures of three potent and three low-activity antibodies bound to Pfs230 domain 1 revealed four distinct epitopes. Highly potent mAbs from natural infection recognized a common conformational epitope that is highly conserved across *P. falciparum* field isolates, while antibodies with negligible TRA derived from natural infection or immunization recognized three distinct sites. Our study provides molecular blueprints describing *P. falciparum* TRA, informed by contrasting potent and non-functional epitopes elicited by natural exposure and vaccination.

INTRODUCTION

Malaria is caused by *Plasmodium* parasites that are transmitted to humans by *Anopheles* mosquitoes. *Plasmodium falciparum* (*P. falciparum*) causes the most fatal form of malaria, accounting for almost all malarial deaths in sub-Saharan Africa and the majority of malarial deaths worldwide (World Health Organization WHO, 2020). In 2021, the WHO recommended the broad implementation of the RTS,S/AS01 malaria vaccine (trade name Mosquirix) among children in areas with moderate to high *P. falciparum* transmission. RTS,S/AS01 is an anti-infection stage vaccine targeting the *P. falciparum* circumsporozoite protein (PfCSP), which is expressed during pre-erythrocytic stages of infection. While a useful tool, efficacy from this vaccine is only partial and rapidly wanes.[1–3] Indications for a modest reduction in efficacy of RTS,S/AS01 against infections with mismatched CSP alleles,[4] and the fact that this vaccine does not prevent onward transmission to mosquitoes, further highlight the potential benefit of transmission-blocking interventions to complement current interventions. Vaccines that reduce infection and/or transmission, and which are amenable to broad deployment in affected communities, are high priority tools for accelerating malaria elimination and eventual eradication.

Transmission-blocking vaccines (TBVs) are designed to elicit a humoral response in humans capable of disrupting parasite uptake and development in mosquitoes, impeding subsequent

 © 2023 The Authors. Published by Elsevier Inc.
This is an open access article under the CC BY license (http://creativecommons.org/licenses/by/4.0/).

CellPress
OPEN ACCESS

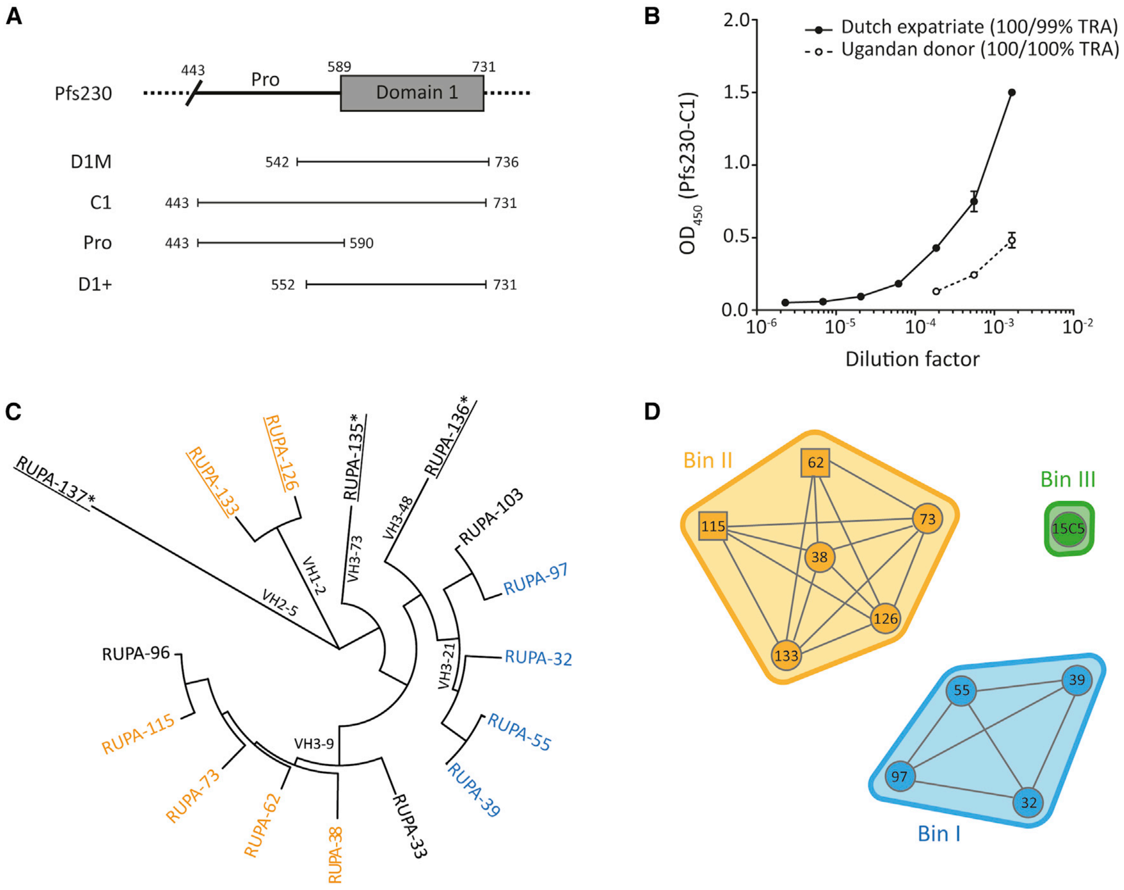

Figure 1. Genetics and binding characteristics of Pfs230-C1 antibodies

(A) Schematic representation of a fragment of Pfs230, focusing on the pro-domain and first of fourteen 6-Cys domains. Domain boundaries are indicated by amino acid numbers. The leading Pfs230-vaccine construct (D1M[14]) and recombinant constructs used in this study (C1, Pro, and D1+) are shown below.

(B) Recognition of Pfs230-C1 by plasma samples from two naturally exposed donors in enzyme-linked immunosorbent assay. Values are means of two technical replicates, and error bars represent the SEM. TRAs of purified total IgG, tested at 1:3 dilution in the presence of complement, from both donors are shown in the legend and are outcomes of two independent SMFAs. The raw SMFA data can be found in Table S2.

(C) Phylogenetic tree of heavy-chain gene sequences, generated with MEGA7 software using default settings.[16] mAbs that were derived from the Ugandan donor (n = 5) are underlined. mAbs that target the pro-domain are indicated with an asterisk. Names are colored according to competition data in (D), while mAbs without available competition data are shown in black.

(D) Epitope bins were determined by competition experiments and are shown as envelopes. Competition between antibodies was tested in two orientations; one antibody coupled to a chip and the other one in solution, and vice versa. Antibodies with data in two directions are shown in circles and those with data in one direction as squares. Competition data were only available for ten of the C1-specific antibodies. See also Figures S1 and S2.

mosquito-to-human transmission.[5–8] During human-to-mosquito transmission, male and female *Plasmodium* gametocytes are ingested during a blood meal and activated in the mosquito midgut to form fertile microgametes and macrogametes, which are exposed to human antibodies that are also present in the blood meal. Multiple studies have identified sexual stage antigens on the gamete surface; vaccine-induced and naturally acquired antibodies can bind to these antigens and block *Plasmodium* fertilization or later sporogonic development.[9,10]

Pfs230 is the target of the most clinically advanced TBV candidate, which is currently being evaluated in a phase 2 clinical trial (NCT03917654). Pfs230 is a 363 kDa secreted protein containing 14 six-cysteine (6-Cys) domains and is important for gamete adherence to human red blood cells and subsequent oocyst formation.[11,12] Transmission-reducing activity (TRA) by antibodies elicited in mice has been found to target the N-terminal region of the protein,[13] and the clinical Pfs230-based candidate immunogen contains the first 6-Cys domain of Pfs230 with a segment of its N-terminal pro-domain (referred to as D1M, amino acids 542–736; Figure 1A).[14] In the vaccine immunogen, this domain is chemically cross-linked to the carrier protein exoprotein A (EPA).[15]

In animal and human trials, monoclonal antibodies (mAbs) have been identified that target Pfs230D1M and possess a range of transmission-reducing potencies.[13,17,18] Previous structural characterizations and epitope-binning assays identified two distinct epitopes on the core Pfs230 domain 1 (D1), which are targeted by mAbs with TRA.[17,18] Importantly, in the human study where participants were immunized with Pfs230D1M-EPA/Alhydrogel, eight of the nine mAbs identified lacked potent TRA and bound to separate but unspecified epitopes, compared with the single functional mAb.[18] While TRA of Pfs230-targeting

mAbs is substantially enhanced by the presence of complement,[14,18–20] why certain high-affinity mAbs of the complement-fixing isotype targeting Pfs230 D1 lack TRA is unclear. We previously demonstrated that Pfs230-targeting polyclonal antibodies purified from the sera of naturally exposed individuals can possess TRA.[21] Prior to this study, no mAbs targeting Pfs230 have been identified from individuals naturally infected with *P. falciparum* malaria. Delineation of epitopes of vaccine-induced and naturally induced mAbs, integrated with a fine-mapping of their inhibitory potencies, has the potential to inform rational immunogen design of a next-generation Pfs230 D1 vaccine to preferentially elicit antibodies with potent TRA.

In this study, we present a multifaceted analysis of Pfs230 D1-targeting human mAbs elicited by natural *P. falciparum* infection. The Pfs230 D1-reactive B cell repertoire is reported for two donors afflicted by recurrent malaria infections who express polyclonal IgG with potent TRA. To elucidate the structural basis of antibody-mediated inhibition across a range of potencies, we determined the crystal structures of six antibodies in complexes with Pfs230 D1. Collectively, our study reveals a detailed structure-function understanding of transmission-blocking activity by antibodies against Pfs230 D1.

RESULTS

Pfs230 monoclonal antibodies identified from naturally exposed donors

We identified two donors with naturally acquired transmission-blocking immunity and serum antibodies against Pfs230-C1 (Figures 1A and 1B): a Dutch expatriate who lived in Central Africa for approximately 30 years (previously donor A[21]) and an 8-year-old Ugandan donor from Tororo, an area with intense malaria transmission in eastern Uganda.[22] We previously showed that Pfs230-specific polyclonal antibodies from the Dutch expatriate blocked transmission when tested at high concentration.[21] To isolate mAbs, memory B cells from these donors were activated, and single cells were sorted using a microfluidic screening device. Single B cells secreting Pfs230-C1-reactive antibodies were identified using two different screening methods (Figures S1A–S1D). Sequencing of positive hits revealed 20 unique sequences that were produced as human IgG1 mAbs. Sixteen of these mAbs bound to Pfs230 C1, three of which (RUPA-135, -136, and -137) bound to the pro-domain of Pfs230 (Figures S1E–S1H). Four mAbs did not bind to Pfs230 C1, suggesting that these represent non-specific hits from initial screening. All but the pro-domain-specific mAbs recognized a conformational epitope on native Pfs230 in western blot (Figure S2A). All Pfs230 C1-specific mAbs recognized Pfs230 on the surface of wild-type (WT) female gametes (Figure S2B). mAbs that reacted with the pro-domain showed cross-reactivity with other antigens in gametocyte extract, suggesting that these either have a promiscuous antigen-binding site or recognized an epitope that is conserved across multiple antigens. The 11 mAbs that were derived from the Dutch expatriate were encoded by *VH3-9* and *VH3-21* genes, whereas the 5 mAbs from the Ugandan donor showed more genetic diversity with 4 different VH genes (Figure 1C; Table S1). Surface plasmon resonance (SPR)-based competition experiments identified two unique bins; bin I contained VH3-21-encoded antibodies from the Dutch expatriate, and bin II contained VH3-9-encoded mAbs from the Dutch expatriate and VH1-2-encoded mAbs from the Ugandan donor (Figure 1D). For the pro-specific mAbs RUPA-135, -136, and -137 and domain 1-specific mAbs RUPA-33, -96, and -103, no competition data were available. 15C5, a humanized analog of the 15C5 mouse mAb that was elicited after immunization with recombinant Pfs230 C1 (residues 443 to 731[23]), was included in the competition experiments. 15C5 is a non-functional mAb occupying bin III and does not compete with mAbs that are in bins I or II (Figure 1D).

Monoclonal antibodies from bin I potently block parasite transmission

To determine whether the identified mAbs blocked parasite transmission to mosquitoes, we tested these at 100 μg/mL in standard membrane feeding assays (SMFAs) with cultured *P. falciparum* NF54 gametocytes and *Anopheles stephensi* mosquitoes. The four mAbs from bin I and RUPA-103—which is genetically similar to these four mAbs and thus likely to belong to the same bin—reduced transmission by more than 80%, whereas none of the other mAbs showed substantial TRA (Figure 2A). The activity did not seem to correlate with binding affinity to the recombinant protein, as some of the mAbs with the highest affinity showed low activity (Figures S1I and S1J). mAbs from bin I displayed complement-dependent TRA (Figure 2B) and had IC_{80} values that ranged between 0.7 and 10.2 μg/mL (Figure 2C). Together, these data suggest that the inhibitory capacity of the Pfs230 mAbs depended on the epitope targeted and the ability to fix complement.

Structural delineation of a potent TRA epitope on Pfs230

To elucidate the structural basis of antibodies with potent TRA acquired following natural infection, we solved three crystal structures of antibody-antigen-binding fragments (Fabs) for RUPA-32, -55, and -97 bound to Pfs230 D1+ (Figure 3A) at resolutions of 2.6, 2.9, and 3.3 Å, respectively (Figures S3A–S3C; Table S3). As observed in previously solved crystal structures of Pfs230 D1 (PDB IDs 6OGH and 7JUM), the 6-Cys core domain of the D1+ protein adopts a mixed β sandwich fold with two disulfide bridges linking residue C593 to C611 and residue C626 to C706. We observed conformational heterogeneity of the N-terminal pro-domain residues 562–576 directly preceding the core 6-Cys domain, which were found packed against the core domain in one of two biological assemblies of the Fab RUPA-55 bound structure but not observed in the other assembly (Figures S3D and S3E), suggesting that these residues may not be necessary for domain folding or binding by potent mAbs.

In agreement with the epitope-binning data (Figure 1D), all three Fabs bound a common antigenic site on Pfs230 D1+. Superposition of the three complexes by structural alignment of the Pfs230 D1+ core domain revealed a highly similar interaction interface (Figure 3B). Indeed, all three Fabs bound nearly identical discontinuous conformational epitopes on Pfs230 D1+, which comprise the following: residues from β strands 1 (amino acids 580–583), 2 (amino acid 585), 3 (amino acids 592–594), and 4 (amino acids 608, 611, and 612); loops 2 (amino acid 584), 4 (amino acids 595, 597, 598, 600–603, and 607), 6 (amino acids 632 and 635), and 12 (amino acids 716 and 719); and the disulfide bridge formed by C593-C611 (Figure 3C). The three

CellPress
OPEN ACCESS

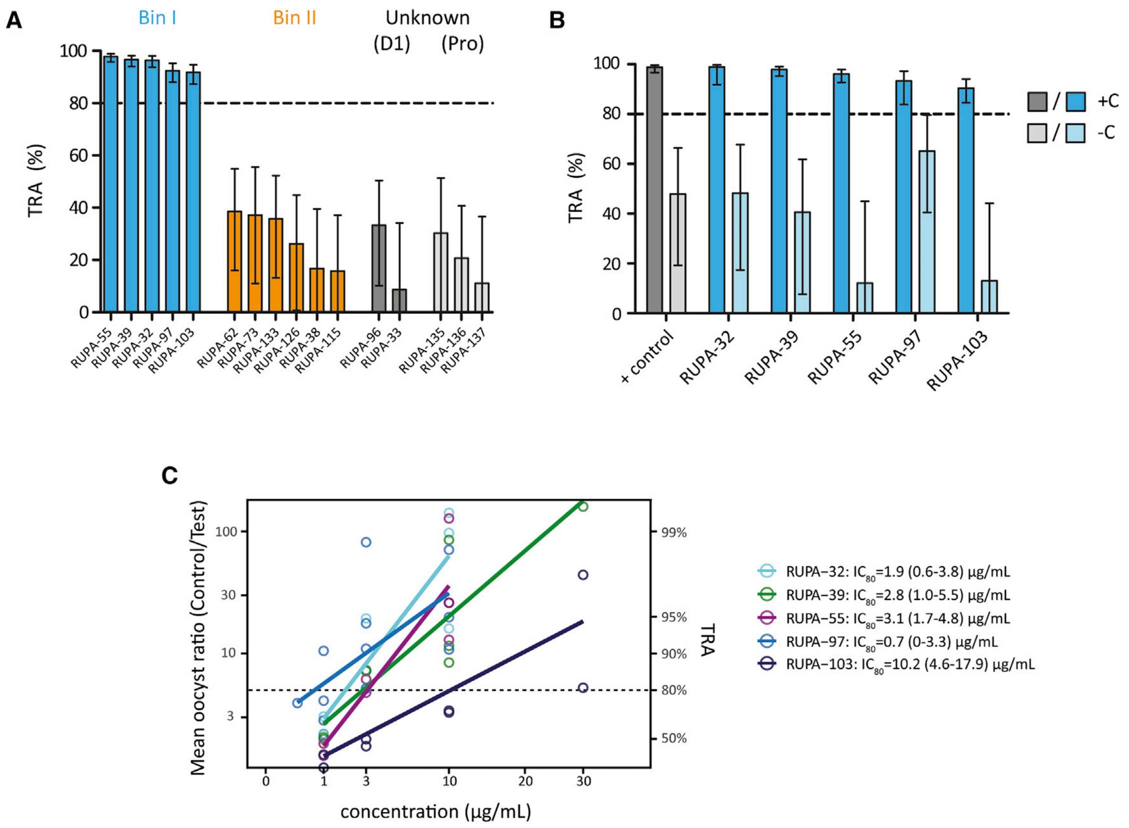

Figure 2. Pfs230-C1 mAbs show a range of functional activity in standard membrane feeding assay

(A) mAbs were tested at 100 μg/mL in parallel in the same membrane feeding experiment. Transmission-reducing activity (TRA) is reduction in oocyst intensity, compared with negative control, and error bars indicate 95% confidence intervals. Antibodies are grouped and colored by domain specificity and epitope bin. No binning data are available for RUPA-103, but it is genetically similar to RUPA-97 (VH and VL amino acid sequence homology >95%) and therefore grouped with other antibodies from bin I.

(B) mAbs that showed >80% TRA in (A) were tested at 100 μg/mL in the presence (+C) or absence (−C) of complement in two independent membrane-feeding experiments, except for RUPA-32 and RUPA-97 that were tested in a single experiment due to limited mAb availability. The complement-dependent rodent α-Pfs230 mAb 2A2[24] was tested at 10 μg/mL and used as positive control. Bars are estimates of the mean from two independent standard membrane feeding experiments, and error bars represent the 95% confidence intervals.

(C) Titrations of active mAbs were tested in multiple independent membrane feeding assays in the presence of complement to calculate IC_{80} values, using linear regression analysis. IC_{80} values with 95% confidence intervals are shown next to graph. Raw SMFA data can be found in Table S2.

antibodies are marginally distinguished by the inclusion of one or two distinct residues present at the periphery of the core epitope on the antigen surface (Figures S4A and S4B). The distinguishing residues are S604 and K614, S578 and N586, and K610, for RUPA-32, -55, and -97, respectively.

Potent antibodies bind broadly across Pfs230 D1 polymorphisms

Sexual stage antigens are typically more genetically conserved, compared with asexual vaccine antigens, yet some non-synonymous single-nucleotide polymorphisms (SNPs) have been observed to affect the TRA of anti-Pfs230 antibodies.[10] We assessed the prevalence of SNPs occurring on Pfs230 D1+ and within the epitopes of RUPA-32, -55, and -97, using sequence data from 7,113 *P. falciparum* field isolates collected from 73 different locations in Africa, Asia, America, and Oceania (MalariaGen[25]) (Table 1). Of the 30 SNPs that induced amino acid changes within Pfs230 D1+, six are located within the epitopes of RUPA-32, -55, and -97 (Figure 3C). Low allele frequencies with no co-occurrences were observed for all six bin I polymorphisms (A583T, 0.022%; T602K, 0.014%; E612K, 0.0072%; V632A, 0.029%; K716N, 0.022%; and N719S, 0.022%), thus indicating strong genetic conservation of this epitope that is recognized by mAbs with potent TRA (Tables 1 and S4).

To evaluate any potential effect on binding by functional antibodies, we assessed the impact of these six low-frequency SNPs on the affinity and kinetics of the three Fabs of RUPA-32, -55, and -97. As a control, we also measured binding of Fab RUPA-38, which binds in a distinct epitope bin on Pfs230 D1 (Figure 1D). As expected, the binding of Fab RUPA-38 was not affected by any of the SNP mutations, compared with WT Pfs230 D1+ binding (Figure 3D). The binding affinities of the Fabs of RUPA-32, -55, and -97 for Pfs230 D1+-mutant constructs V632A, K716N, and N719S did not change substantially relative to the wild-type Pfs230 D1+ construct (Figure 3D). Marginal decreases in binding affinities were detected for the other three SNPs, but importantly, all binding affinities remained in

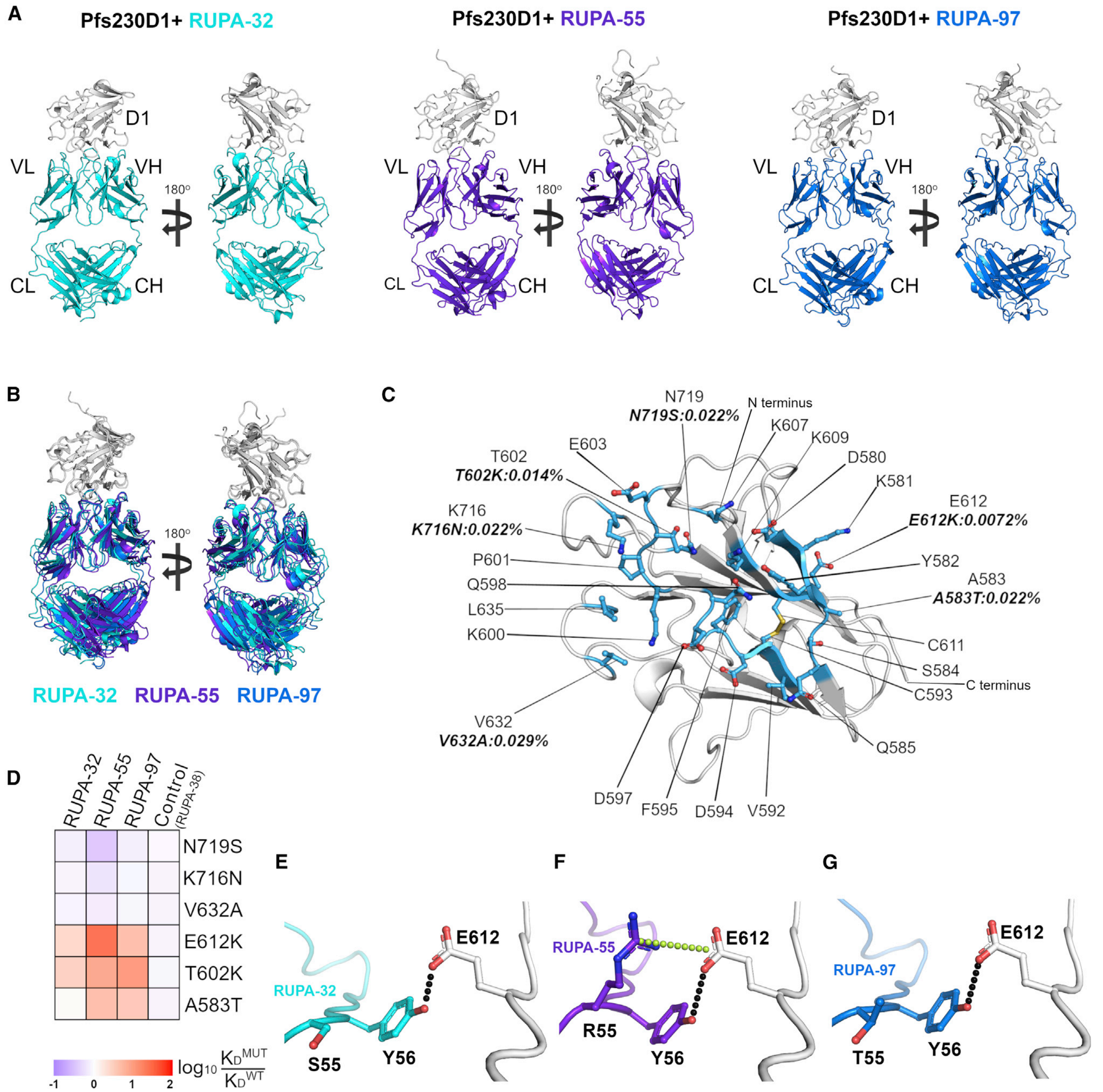

Figure 3. Structural delineation of a high-potency transmission-blocking epitope on Pfs230 D1+

(A) Overall structures of Pfs230 D1+ (white) bound by Fabs RUPA-32, -55, and -97 (cyan, purple, and blue, respectively).

(B) Superposition of all three Fab-antigen structures from (A) structurally aligned using Pfs230 D1+.

(C) The common epitope on Pfs230 D1+ between all three Fabs colored in marine blue. Epitope residues are labeled, and side chains are shown as sticks. SNPs occurring within the epitope are labeled along with their allele frequency (Table 1).

(D) Relative effect of SNP mutations on the binding constants of Fabs RUPA-32, -55, and -97. RUPA-38 binds to a different epitope on Pfs230 D1+ and was used as a control (Table S5).

(E–G) Hydrogen bonds (black) and a salt bridge (yellow) formed between E612 on Pfs230 and residues on RUPA-32, -55, and -97, respectively. The salt bridge is drawn between resonant charge centers of the arginine and glutamate side chains. See also Figures S3–S5.

the low nanomolar range for all SNPs indicating strong binding to all Pfs230 polymorphisms for this set of high-potency inhibitory antibodies (Table S5). Notably, the largest binding affinity perturbation was observed for RUPA-55 and the E612K mutation, with K_D values of 2.4 and 58 nM for the WT and E612K construct, respectively (Figure 3D; Table S5). On Pfs230, E612 interacts with the complementarity-determining region (CDR)-H2 of RUPA-32, -55, and -97 through an H bond mediated by a germline-encoded tyrosine residue at position 56 (Figures 3E–3G). In addition, a salt bridge is mediated by

CellPress
OPEN ACCESS

Table 1. Coding SNPs are present in Pfs230 D1+ amino acids 552–731

Residue	Coding SNP	Allele frequency (%)	RUPA-32	RUPA-55	RUPA-97	4F12	RUPA-38	15C5	LMIV 230-01	LMIV 230-02
561	D561N	0.014	–	–	–	–	■	–	–	–
574	V574I	0.014	–	–	–	–	–	–	■	–
575	S575P	0.014	–	–	–	–	–	–	■	–
583	A583T	0.022	■	■	■	■	–	–	–	–
602	T602K	0.014	■	■	■	■	–	–	–	–
605	G605S	94.4	–	–	–	–	–	–	–	–
612	E612K	0.0072	■	■	■	–	–	–	■	–
632	V632A	0.029	■	■	■	–	–	–	–	–
637	D637N	0.014	–	–	–	–	–	–	–	–
644	K644Q	0.014	–	–	–	–	■	–	–	–
652	T652R	2.1	–	–	–	–	–	–	–	–
654	E654K	0.014	–	–	–	–	–	–	–	–
655	E655V	0.35	–	–	–	–	–	–	■	–
656	T656N	0.61	–	–	–	–	–	–	■	–
661	K661N	21.6	–	–	–	–	–	–	–	–
	K661T	0.029	–	–	–	–	–	–	–	–
665	K665Q	0.0072	–	–	–	–	–	■	–	–
675	T675K	0.0072	–	–	–	–	–	–	–	■
687	V687I	0.036	–	–	–	–	–	–	–	–
697	H697Q	0.014	–	–	–	–	–	■	–	–
699	A699T	0.33	–	–	–	–	–		■	–
701	V701M	0.0072	–	–	–	–	–		■	–
713	D713Y	2.9	–	–	–	–	–	–	–	–
714	D714G	0.029	–	–	–	–	–	–	–	–
	D714N	0.014	–	–	–	–	–	–	–	–
715	N715K	0.022	–	–	–	–	–	–	–	–
716	K716N	0.022	■	■	■	–	–	–	–	–
719	N719S	0.022	■	■	■	–	–	–	–	–
726	Y726H	0.0072	–	–	–	–	–	–	■	–
727	V727I	0.014	–	–	–	–	–	–	–	–

Coding SNPs occurring within the epitopes reported for all known antibody-antigen structures are indicated with black squares. PDB IDs for Fab 4F12 and scFv LMIV230-01 are 6OHG and 7JUM, respectively.

RUPA-55 CDR-H2 residue R55 and E612 on Pfs230 (Figure 3F). An electrostatic repulsion introduced by the E612K mutation likely explains why binding by RUPA-55 was more perturbed, compared with RUPA-32 and -97 (Figure 3D; Table S6).

Germline-encoded residues contribute to potent Pfs230 D1 epitope recognition

In this study, antibodies with potent TRA arose from one of the two donors and utilized a common heavy-chain germline lineage (IGHV3-21), which was diversified across two kappa light-chain germline lineages. Our three structures captured this kappa light-chain diversity, wherein RUPA-32 and -97 arose from the IGKV3-11 germline lineage, while RUPA-55 arose from the IGKV3-15 germline lineage. Comparison of the variable gene-encoded CDR-H2, -K1, and -K3 sequences that contact the Pfs230 antigen indicates a high degree of conservation with the corresponding germline sequences (Figure S5A). Furthermore, examination of the paratopes revealed analogous structural configurations (Figure S5B), which facilitate a conserved network of H bonds (Figures S5C–S5E; Table S6). Indeed, several germline-encoded residues were found to be involved in side-chain-mediated H bonds. For example, in CDR-K1, the side chain and backbone of germline-encoded residues S28 and S30 found in RUPA-32, -55, and -97 were observed to form conserved H bonds with the D597 and K600 side chains of Pfs230 (Figure S5C–S5E, column 3; Table S6). One point of difference was observed in CDR-H3, where the peak buried surface area (BSA) contributions of RUPA-32 and RUPA-97 occur at position 100 but was offset to position 100a (Kabat numbering) in the RUPA-55 structure (Figure S5B, yellow panel). These offset CDR-H3 loops adopted equivalent H-bonding networks, wherein the CDR-H3 residues with the largest BSA contributions (M100, L100a, and L100 for RUPA-32, -55, and -97, respectively) all formed a backbone-mediated H bond with the side chain of Pfs230 Q598 (Figures S5C–S5E, column 2; Table S6). The similar BSA signatures of all paratope CDRs, except for CDR-H3, illustrate the unique structural characteristics often observed for the CDR-H3 loop.[26]

Delineation of a low-potency epitope recognized by a naturally acquired mAb

To understand why some mAbs against Pfs230 D1 acquired following natural infection lack potent TRA, we expanded our analysis to a low-potency mAb from bin II by determining the crystal structure of the Fab of RUPA-38 bound to Pfs230 D1+ (Figure 4A) at a resolution of 2.1 Å (Figure S3F; Table S3). RUPA-38 bound to a discontinuous conformational epitope on Pfs230 D1+, composed of residues from β strands 6, 8, 10, and 11 and loops 1, 7, 8, and 13 (Figure 4B). Pfs230 D1+ residues involved in the interaction with RUPA-38 were found to be fully distinct from the residues that form the potent TRA epitope of bin I mAbs bound by RUPA-32, -55, and -97 (Figures 3C and 4B).

Non-inhibitory mAbs bind the C terminus of recombinant Pfs230 D1 immunogens

To further explore why some mAbs lack potent TRA, we determined the crystal structures of two additional antibody-antigen complexes, using Fabs derived from previously reported studies with recombinant Pfs230 immunogens. The first mAb, 15C5, did not recognize Pfs230 on the surface of female gametes (Figure S2C) and was previously described to lack detectable TRA at 375 μg/mL by SMFA. The second mAb, LMIV230-02, was identified in the same study as LMIV230-01 from an individual immunized with Pfs230 D1 (Pfs230D1M-EPA/Alhydrogel), but unlike LMIV230-01, LMIV230-02 showed negligible activity with only ~59% TRA at 1,000 μg/mL in SMFA.[18] After generating Fabs, we determined the crystal structures of Fab 15C5 and Fab LMIV230-02 bound to Pfs230 D1+ (Figures 4C and 4D) at resolutions of 3.3 and 2.0 Å, respectively (Figures S3G and S3H; Table S3). Neither epitope overlapped with any protective epitopes described here and so far. Notably, the epitopes of both Fabs are directly adjacent to one another, with Pfs230 residue I672 shared between them (Figure 4E). While both Fabs were bound to a discontinuous conformational epitope on Pfs230 D1+, both epitopes primarily consist of loops proximal to the C terminus of D1.

We hypothesized that the low/absent TRA of 15C5 and LMIV230-02 might be explained by their binding to a Pfs230 epitope either fully or partially inaccessible on the native protein. Indeed, similar to 15C5, LMIV230-02 was also previously shown to be incapable of labeling the surfaces of live *P. falciparum* gametes in immunofluorescent assays.[18] Testing this hypothesis, we employed a theoretical modeling approach to examine the accessibility of the 15C5 and LMIV230-02 epitopes on a structural model of a Pfs230 fragment containing domains 1 and 2 (residues 552–889; referred to as Pfs230 D1D2). We relied on theoretical models since, up to this point, recombinant Pfs230 fragments other than D1 were unavailable partly because of inadequate expression and purification strategies that preserve proper folding. To evaluate the theoretical disposition of D2 relative to D1, our highest resolution crystal structure of Pfs230 D1+ (Figure 4F) was compared with the theoretical models of Pfs230 D1D2 generated by RoseTTAFold[27] (Figure 4G), AlphaFold2,[28] I-Tasser,[29] and RaptorX[30] (Figure S6). All Pfs230 D1D2 models have domain 2 in the same orientation relative to D1 (Figures 4G, S6A, and S6B). Fabs 15C5 and LMIV230-02 overlapped with domain 2 in all the theoretical models (Figures 4H, 4I, S6C, and S6D), suggesting that the epitopes of both antibodies may be sterically occluded by domain 2, which could contribute to their lack of potent TRA.

DISCUSSION

Here, we are characterizing a diverse panel of Pfs230 D1-targeting mAbs and presenting a multifaceted structure-function analysis describing the mechanism of TRA against this leading TBV antigen. By examining mAbs that were elicited from natural *P. falciparum* infections, we identified one family of antibodies that target a highly conserved epitope on Pfs230 D1 and possess the most potent TRA described for human Pfs230 antibodies to date. By expanding our analysis to previously identified non-functional mAbs (15C5 and LMIV230-02) and comparing them with the only other two functional mAbs previously reported (4F12[14,17] and LMIV230-01[18]), we have identified three general epitope bins on Pfs230 D1 (Figures 5A and 5B). Together, our structural studies generate molecular insight into functional and non-functional epitopes on Pfs230 and provide a roadmap for improved immunogen design.

Complement-mediated killing is the putative mechanism of TRA observed for anti-Pfs230 D1 antibodies,[14,19,20] including the functional mAbs that we have identified in this study and those elicited by the Pfs230D1M-EPA/Alhydrogel vaccine candidate.[18,20] All mAbs in the present study were expressed as complement-fixing human IgG1 antibodies, which allowed us to establish other factors that affect potency, such as affinity and targeted epitope. Our comparative analysis indicates that the potent TRA mAbs derived from natural infection, which bind at epitope bin I, have superior TRA to rh4F12[17] (Figure 5C), even though they bind to a mutually inclusive epitope with a similar antibody angle of approach (Figures S4C and 4D). This difference may be attributed to the stronger affinity of these mAbs derived from natural infection, compared with rh4F12 (Table S5). Prior to our study, LMIV230-01 was the only other human D1-directed mAb reported to possess TRA.[18] By comparison, the epitope bin II mAb RUPA-38 possesses sub-nanomolar binding affinity for Pfs230 D1+ but lacks detectable TRA (Figure 5C). While the RUPA-38 and LMIV230-01 competitively bind to Pfs230 D1 with partially overlapping epitopes, the angles of approach differ considerably (Figures 5A, S4E, and S4F). Additionally, unlike all other bin II mAbs, LMIV230-01 does possess modest complement-dependent TRA, with an observed IC_{80} value similar to that of rh4F12 (Figure 5C), indicating high affinity and complement-fixing subclass together may not be sufficient to impart potent TRA. Instead, the angle of antibody approach toward Pfs230, in addition to high affinity and complement-fixing subclass, may be critical for functional activity. Similarly, recent studies in an unrelated system have also observed that antibody angle of approach toward CD20 can establish an optimal geometry necessary for complement recruitment.[31,32] Altogether, our multifaceted structure-function analysis strongly emphasizes the diversity of determinants of complement-dependent antibody function regarding the mechanism of action of Pfs230 D1-targeting mAbs with potent TRA.

Structure-based immunogen design continues to enable the elicitation of potent humoral responses that target biologically relevant epitopes across pathogens. A recent successful example of this approach, stemming from previous advances

CellPress
OPEN ACCESS

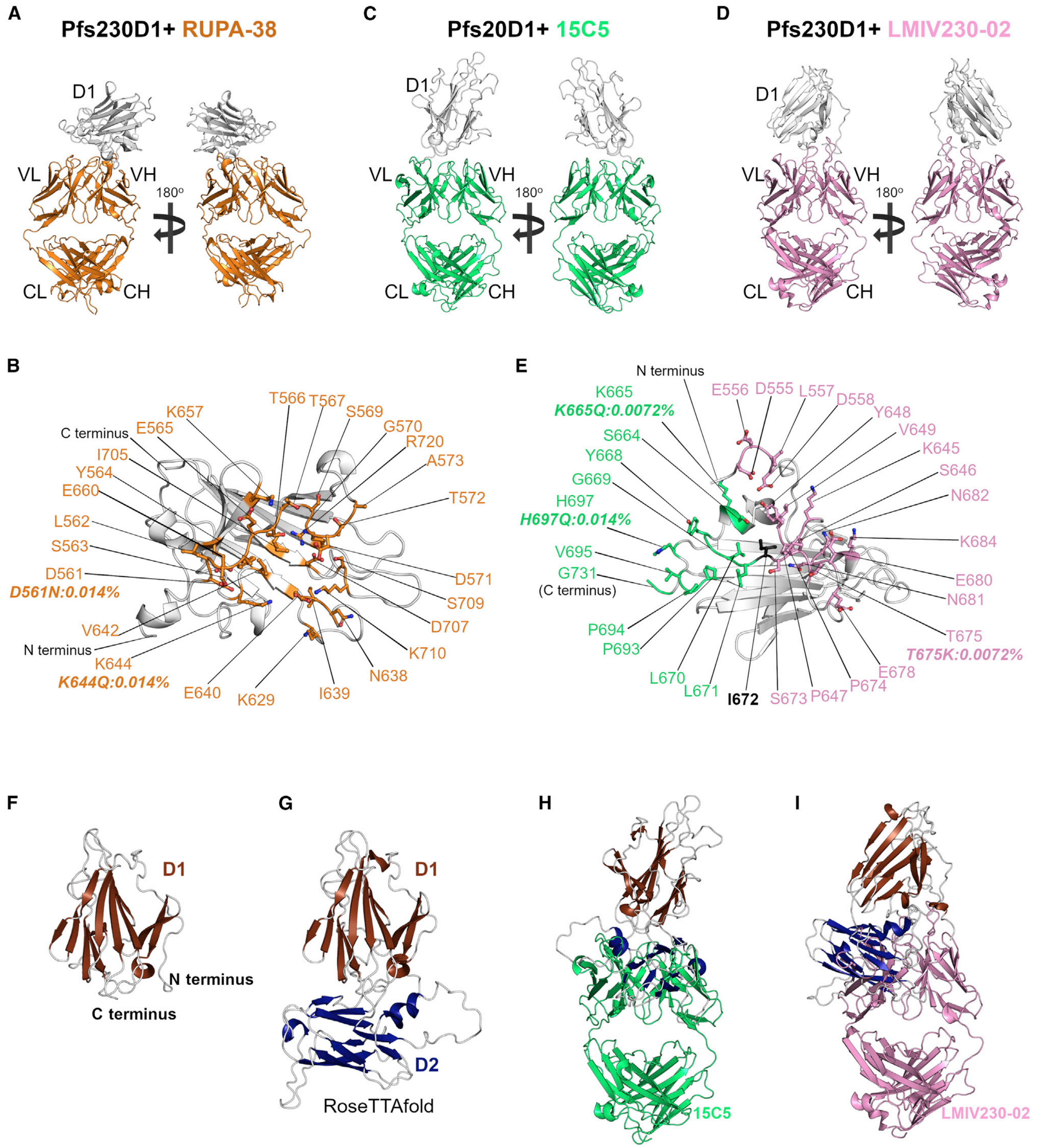

Figure 4. Structural delineation of antibodies bound to low-potency Pfs230 D1+ epitopes

(A) Overall structure of Pfs230 D1+ (gray) bound by Fab of RUPA-38 (orange).

(B–D) The RUPA-38 epitope on Pfs230 D1+ is colored in orange. Overall structures of Pfs230 D1+ (white) bound by (C) Fab 15C5 (green) and (D) Fab LMIV230-02 (pink).

(E) Adjacent (green and pink) and partially overlapping (black) residues on Pfs230 D1+ are shown. All epitope residues from (B) and (E) are labeled, and side chains are shown as stick representation. SNPs occurring within the epitopes are labeled along with their allele frequency (Table 1).

(F–I) The high-resolution structure of Pfs230 D1+ (2.0 Å) from the LMIV230-02 complex and (G) a theoretical model of Pfs230 domains 1 (D1) and 2 (D2) (residues 552–889) generated using RoseTTAfold.[27] Superposition of Fabs (H) 15C5 and (I) LMIV230-02 on the model of Pfs230 D1D2, based on the binding sites shown in (C) and (D), shows clear clashing between the Fabs and D2. See also Figures S3 and S6.

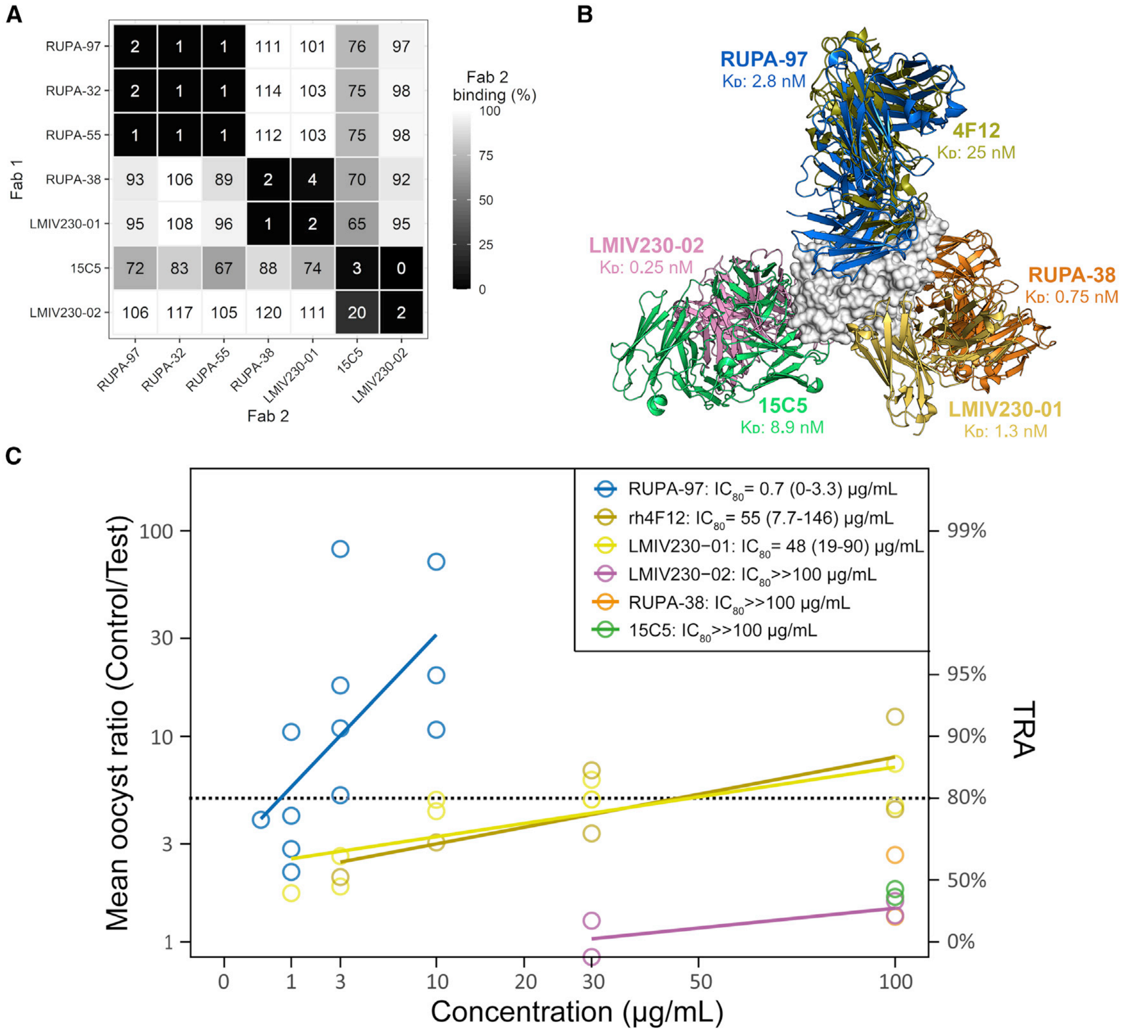

Figure 5. Epitope delineation of known Pfs230-D1-binding antibodies

(A) Heatmap of binding competition experiments. High signal responses for the second binding event (white) represent low competition, whereas low signal responses (black) correspond to high competition.

(B) Superposition of all known structures of mAbs bound to Pfs230 D1. RUPA-97 serves as an exemplar of the bin I mAbs from natural infection. Binding affinity measured by biolayer interferometry is also indicated in Table S5. PDB IDs for Fab 4F12 and scFv LMIV230-01 are 6OHG and 7JUM, respectively.

(C) mAbs from different epitope bins were tested in multiple independent SMFAs to calculate IC_{80} values (μg/ml), using linear regression analysis. IC_{80} values with 95% confidence intervals are shown in inset. TRA, transmission-reducing activity (reduction in oocyst intensity, compared with negative control). Colors correspond to (B). Raw SMFA data can be found in Table S2.

in the field,[33,34] is the leading SARS-CoV-2 spike protein immunogen, which possesses two designed proline mutations that stabilise the spike in its pre-fusion conformation to preferentially elicit antibody responses against the most potent epitopes present in this state.[35] The discordance between natural and subunit-based humoral responses uncovered in our study suggests that Pfs230 D1 as a target may lend itself to further immunogen improvements. Indeed, mAb LMIV230-01 derived from the Pfs230D1M-EPA clinical trial was the only one of nine mAbs found to possess moderate TRA, as shown in a recent study.[18] Further, non-functional mAbs LMIV230-02 and 15C5 elicited from Pfs230 D1 subunit vaccines bind to epitopes at the C terminus of Pfs230 D1 (Figures S4G and S4H). Theoretical models indicate that these epitopes could be partially buried at the interface between Pfs230 D1 and D2 in the native protein, which explains their lack of TRA and clarifies the deficiency of LMIV230-02 to recognize native Pfs230 in immunofluorescent assays.[18] No antibodies derived from natural infection in our dataset bound to this site. Thus, structural studies characterizing antibody responses elicited by either natural infection or immunizations with subunit vaccines have the potential to inform rational immunogen design efforts for immunofocusing the most potent epitopes. By example, broadly neutralizing mAbs elicited during chronic HIV-1 infection have been found to target conserved areas on the HIV-1 Env protein such as the trimer apex,[36,37] CD4 interface,[38,39] gp41 interface,[40] and glycan-dependent interfaces.[41,42] By contrast, the non-neutralizing mAbs elicited from recombinant HIV-1 Env trimer subunit vaccines[43] often

target non-native epitopes, such as the trimer base,[44] exposed V3 loop,[45,46] and artificial glycan holes.[47] Immunofocusing through the introduction of N-linked glycosylation sites or by epitope scaffolding has shown promise for HIV-1 Env,[48–50] influenza hemagglutinin,[51] and RSV F immunogens[52,53] and may be viable strategies that should be explored for a next-generation Pfs230D1-based immunogen to focus away from an exposed C terminus and more toward the exquisitely potent bin I epitope delineated in detail in this study. Antigen scaffolding of different TBV immunogens also offers another opportunity to prioritize epitopes with potent TRA, which potentially elicit an additive or synergistic humoral response and should be explored in future studies.

Three sexual stage antigens, Pfs230, Pfs48/45, and Pfs25, are currently in active phase 2 (NCT03917654), phase 1 (NCT04862416), and phase 1 (NCT04271306) clinical trials, respectively. Accordingly, Pfs230 is the most clinically advanced TBV candidate, with Pfs230D1M-EPA/Alhydrogel showing superior clinical efficacy to Pfs25 in comparative studies in humans and *Rhesus macaques*.[20,54] Importantly, the humoral response to gametocyte TBV immunogens such as Pfs230 may be reinforced by prior and/or subsequent natural infection.[55] Humoral responses against Pfs230 are common in malaria endemic regions,[56,57] and we have recently shown that affinity-purified Pfs230-specific antibodies can indeed block transmission.[21] LMIV230-01 is the only other human-derived mAb reported to date with observed TRA[18] (Figure 5C) and is encoded by *IGHV1-69* and *IGKV1-5*, which were the most common germline genes identified from the memory B cells sequences obtained from the eight vaccinees in Coelho et al.[18] *IGHV1-69* and *IGKV1-5* were not common to any of the mAbs identified in this study. Together, these considerations highlight a need for the discovery of the most potent antibody clones directed toward Pfs230 D1. Immunogen designs capable of preferentially eliciting such potent antibodies will have the potential to aid ongoing malaria eradication efforts. In summary, the data presented here provide a compelling rationale for further analysis of mAbs derived from naturally exposed populations to better understand responses to current TBV candidates under clinical evaluation and inform the design of next-generation TBV immunogens.

Limitations of the study

This study presents the most potent Pfs230 D1-directed mAbs described to date, with structural and functional contrast to several lower-potency mAbs; however, it is limited in terms of the scope of participants. Here, we took advantage of B cells derived from two individuals who were repeatedly exposed to malaria in order to examine the immune repertoire of antibodies targeting Pfs230 in much greater detail. While we have identified mAbs with potent TRA, the scarcity of individuals with naturally acquired transmission-blocking immunity may stem from low circulating antibody concentrations that are insufficient to block transmission. Indeed, while the mAbs in the study were isolated from two donors, all potent mAbs, as well as the non-functional RUPA-38, were derived from a single donor (the Dutch expatriate). Future studies with larger cohort sizes may clarify the epidemiological determinants of humoral transmission-blocking immunity by examining the interplay between participant demographics, germline usages, and extent of parasite exposure. Furthermore, while this study focused exclusively on Pfs230 D1 and its pro-domain, further molecular characterization of potent and non-functional antibodies directed to the other 13 domains in full-length Pfs230 will enlarge our understanding of *P. falciparum* biology and potentially enable expanded vaccine design efforts.

STAR★METHODS

Detailed methods are provided in the online version of this paper and include the following:

- KEY RESOURCES TABLE
- RESOURCE AVAILABILITY
  - Lead Contact
  - Materials Availability
  - Data and Code Availability
- EXPERIMENTAL MODELS AND SUBJECT DETAILS
  - Donor selection and PBMC collection
  - Human cell line culture
- METHOD DETAILS
  - Single B-cell screening and recovery
  - Single B-cell sequencing, bioinformatic analysis and antibody expression
  - Affinity measurements and epitope binning
  - Enzyme-linked immunosorbent assay (ELISA)
  - Western blot
  - Surface immunofluorescence assay (SIFA)
  - Standard membrane feeding assays (SMFA)
  - Fab and antigen expression and purification
  - Crystallization and structure determination
  - Single nucleotide polymorphism detection
  - Biolayer interferometry
  - *In silico* structure modeling
- QUANTIFICATION AND STATISTICAL ANALYSIS

SUPPLEMENTAL INFORMATION

Supplemental information can be found online at https://doi.org/10.1016/j.immuni.2023.01.013.

ACKNOWLEDGMENTS

We would like to thank Jonathan Mandel for assistance with ELISAs; Rianne Stoter and Wiebe Kooijman for parasite culture; Laura Pelser, Astrid Pouwelsen, Jacqueline Kuhnen, Jolanda Klaassen, and Saskia Mulder for mosquito dissection; Karin Koolen for assistance with SIFA experiments; and Greg Wasney for assistance with biolayer interferometry. We are also grateful to Michael Theisen and Susheel Singh for providing the Pfs230-Pro construct. This work was supported by the Canadian Institutes of Health Research project grant no. 428410 (to J.-P.J.). This work was further funded by PATH's Malaria Vaccine Initiative and the Bill & Melinda Gates Foundation (grant no. OPP1170236). Fieldwork leading to the identification of the Ugandan donor was supported by the National Institute of Allergy and Infectious Diseases (NIAID) as part of the International Centers of Excellence in Malaria Research (ICEMR) program (U19AI089674) with laboratory support from NIAID (R01AI093615 and K24AI113002 to M.E.F.). D.I. is supported by a Restracomp fellowship from the Hospital for Sick Children. T.B. is supported by the European Research Council (ERC-CoG 864180; QUANTUM). M.M.J. is supported by the Netherlands Organisation for Scientific Research (Vidi fellowship NWO project number 192.061). J.-P.J. is supported by the CIFAR Azrieli Global Scholar program, the Ontario Early Researcher Award program, and the Canada Research

Chair program. The BLI instrument was accessed at the Structural and Biophysical Core Facility, The Hospital for Sick Children, supported by the Canada Foundation for Innovation and Ontario Research Foundation. X-ray diffraction experiments were performed at GM/CA@APS, which have been funded in whole or in part with federal funds from the National Cancer Institute (ACB-12002) and the National Institute of General Medical Sciences (AGM-12006). The Eiger 16M detector was funded by an NIH–Office of Research Infrastructure Programs High-End Instrumentation grant (1S10OD012289-01A1). This research used resources of the Advanced Photon Source, a U.S. Department of Energy (DOE) Office of Science user facility operated for the DOE Office of Science by Argonne National Laboratory under contract DE-AC02-06CH11357.

AUTHOR CONTRIBUTIONS

Experimental design was collaborative between all authors. Experiments were conducted by D.I., A.F.-G., K.T., M.v.d.V.-B., G.-J.v.G., J.N., A.S., M.d.B., J.B., J.R., M.S., S.V., L.K., S.D., S.-M.L., M.R.K., M.E.F., P.J., and B.G. The manuscript was written by D.I., T.B., M.M.J., and J.-P.J. and edited by all authors.

DECLARATION OF INTERESTS

The authors declare no competing interests.

Received: August 16, 2022
Revised: November 4, 2022
Accepted: January 12, 2023
Published: February 14, 2023

REFERENCES

1. Hogan, A.B., Winskill, P., and Ghani, A.C. (2020). Estimated impact of RTS,S/AS01 malaria vaccine allocation strategies in sub-Saharan Africa: a modelling study. PLoS Med. *17*, e1003377. https://doi.org/10.1371/journal.pmed.1003377.
2. Chandramohan, D., Zongo, I., Sagara, I., Cairns, M., Yerbanga, R.S., Diarra, M., Nikièma, F., Tapily, A., Sompougdou, F., Issiaka, D., et al. (2021). Seasonal malaria vaccination with or without seasonal malaria chemoprevention. N. Engl. J. Med. *385*, 1005–1017. https://doi.org/10.1056/NEJMoa2026330.
3. Datoo, M.S., Natama, M.H., Somé, A., Traoré, O., Rouamba, T., Bellamy, D., Yameogo, P., Valia, D., Tegneri, M., Ouedraogo, F., et al. (2021). Efficacy of a low-dose candidate malaria vaccine, R21 in adjuvant Matrix-M, with seasonal administration to children in Burkina Faso: a randomised controlled trial. Lancet *397*, 1809–1818. https://doi.org/10.1016/S0140-6736(21)00943-0.
4. Neafsey, D.E., Juraska, M., Bedford, T., Benkeser, D., Valim, C., Griggs, A., Lievens, M., Abdulla, S., Adjei, S., Agbenyega, T., et al. (2015). Genetic diversity and protective efficacy of the RTS,S/AS01 malaria vaccine. N. Engl. J. Med. *373*, 2025–2037. https://doi.org/10.1056/NEJMoa1505819.
5. Draper, S.J., Sack, B.K., King, C.R., Nielsen, C.M., Rayner, J.C., Higgins, M.K., Long, C.A., and Seder, R.A. (2018). Malaria vaccines: recent advances and new horizons. Cell Host Microbe *24*, 43–56. https://doi.org/10.1016/j.chom.2018.06.008.
6. Alonso, P.L., Brown, G., Arevalo-Herrera, M., Binka, F., Chitnis, C., Collins, F., Doumbo, O.K., Greenwood, B., Hall, B.F., Levine, M.M., et al. (2011). A research agenda to underpin malaria eradication. PLoS Med. *8*, e1000406. https://doi.org/10.1371/journal.pmed.1000406.
7. Duffy, P.E. (2021). Transmission-blocking vaccines: harnessing herd immunity for malaria elimination. Expert Rev. Vaccines *20*, 185–198. https://doi.org/10.1080/14760584.2021.1878028.
8. Carter, R., and Chen, D.H. (1976). Malaria transmission blocked by immunisation with gametes of the malaria parasite. Nature *263*, 57–60. https://doi.org/10.1038/263057a0.
9. Duffy, P.E., and Patrick Gorres, J. (2020). Malaria vaccines since 2000: progress, priorities, products. npj Vaccines *5*, 48. https://doi.org/10.1038/s41541-020-0196-3.
10. de Jong, R.M., Meerstein-Kessel, L., Da, D.F., Nsango, S., Challenger, J.D., van de Vegte-Bolmer, M., van Gemert, G.J., Duarte, E., Teyssier, N., Sauerwein, R.W., et al. (2021). Monoclonal antibodies block transmission of genetically diverse Plasmodium falciparum strains to mosquitoes. npj Vaccines *6*, 101. https://doi.org/10.1038/s41541-021-00366-9.
11. Gerloff, D.L., Creasey, A., Maslau, S., and Carter, R. (2005). Structural models for the protein family characterized by gamete surface protein Pfs230 of Plasmodium falciparum. Proc. Natl. Acad. Sci. USA *102*, 13598–13603. https://doi.org/10.1073/pnas.0502378102.
12. Eksi, S., Czesny, B., van Gemert, G.J., Sauerwein, R.W., Eling, W., and Williamson, K.C. (2006). Malaria transmission-blocking antigen, Pfs230, mediates human red blood cell binding to exflagellating male parasites and oocyst production. Mol. Microbiol. *61*, 991–998. https://doi.org/10.1111/j.1365-2958.2006.05284.x.
13. Miura, K., Takashima, E., Pham, T.P., Deng, B., Zhou, L., Huang, W.C., Diouf, A., Gebremicale, Y.T., Tachibana, M., Ishino, T., et al. (2022). Elucidating functional epitopes within the N-terminal region of malaria transmission blocking vaccine antigen Pfs230. NPJ Vaccines *7*, 4. https://doi.org/10.1038/s41541-021-00423-3.
14. MacDonald, N.J., Nguyen, V., Shimp, R., Reiter, K., Herrera, R., Burkhardt, M., Muratova, O., Kumar, K., Aebig, J., Rausch, K., et al. (2016). Structural and immunological characterization of recombinant 6-cysteine domains of the Plasmodium falciparum sexual stage protein Pfs230. J. Biol. Chem. *291*, 19913–19922. https://doi.org/10.1074/jbc.M116.732305.
15. Burkhardt, M., Reiter, K., Nguyen, V., Suzuki, M., Herrera, R., Duffy, P.E., Shimp, R., MacDonald, N.J., Olano, L.R., and Narum, D.L. (2019). Assessment of the impact of manufacturing changes on the physicochemical properties of the recombinant vaccine carrier ExoProtein A. Vaccine *37*, 5762–5769. https://doi.org/10.1016/j.vaccine.2018.09.037.
16. Kumar, S., Stecher, G., and Tamura, K. (2016). MEGA7: molecular evolutionary genetics analysis version 7.0 for bigger datasets. Mol. Biol. Evol. *33*, 1870–1874. https://doi.org/10.1093/molbev/msw054.
17. Singh, K., Burkhardt, M., Nakuchima, S., Herrera, R., Muratova, O., Gittis, A.G., Kelnhofer, E., Reiter, K., Smelkinson, M., Veltri, D., et al. (2020). Structure and function of a malaria transmission blocking vaccine targeting Pfs230 and Pfs230-Pfs48/45 proteins. Commun. Biol. *3*, 395. https://doi.org/10.1038/s42003-020-01123-9.
18. Coelho, C.H., Tang, W.K., Burkhardt, M., Galson, J.D., Muratova, O., Salinas, N.D., Alves E Silva, T.L., Reiter, K., MacDonald, N.J., Nguyen, V., et al. (2021). A human monoclonal antibody blocks malaria transmission and defines a highly conserved neutralizing epitope on gametes. Nat. Commun. *12*, 1750. https://doi.org/10.1038/s41467-021-21955-1.
19. Quakyi, I.A., Carter, R., Rener, J., Kumar, N., Good, M.F., and Miller, L.H. (1987). The 230-kDa gamete surface protein of Plasmodium falciparum is also a target for transmission-blocking antibodies. J. Immunol. *139*, 4213–4217.
20. Healy, S.A., Anderson, C., Swihart, B.J., Mwakingwe, A., Gabriel, E.E., Decederfelt, H., Hobbs, C.V., Rausch, K.M., Zhu, D., Muratova, O., et al. (2021). Pfs230 yields higher malaria transmission-blocking vaccine activity than Pfs25 in humans but not mice. J. Clin. Invest. *131*, e146221. https://doi.org/10.1172/JCI146221.
21. Stone, W.J.R., Campo, J.J., Ouédraogo, A.L., Meerstein-Kessel, L., Morlais, I., Da, D., Cohuet, A., Nsango, S., Sutherland, C.J., van de Vegte-Bolmer, M., et al. (2018). Unravelling the immune signature of Plasmodium falciparum transmission-reducing immunity. Nat. Commun. *9*, 558. https://doi.org/10.1038/s41467-017-02646-2.
22. Kamya, M.R., Arinaitwe, E., Wanzira, H., Katureebe, A., Barusya, C., Kigozi, S.P., Kilama, M., Tatem, A.J., Rosenthal, P.J., Drakeley, C., et al. (2015). Malaria transmission, infection, and disease at three sites with varied transmission intensity in Uganda: implications for malaria control. Am. J. Trop. Med. Hyg. *92*, 903–912. https://doi.org/10.4269/ajtmh.14-0312.

23. Lee, S.M., Plieskatt, J., Krishnan, S., Raina, M., Harishchandra, R., and King, C.R. (2019). Expression and purification optimization of an N-terminal Pfs230 transmission-blocking vaccine candidate. Protein Expr. Purif. *160*, 56–65. https://doi.org/10.1016/j.pep.2019.04.001.

24. Roeffen, W., Geeraedts, F., Eling, W., Beckers, P., Wizel, B., Kumar, N., Lensen, T., and Sauerwein, R. (1995). Transmission blockade of Plasmodium falciparum malaria by anti-Pfs230-specific antibodies is isotype dependent. Infect. Immun. *63*, 467–471. https://doi.org/10.1128/iai.63.2.467-471.1995.

25. MalariaGEN, Ahouidi, A., Ali, M., Almagro-Garcia, J., Amambua-Ngwa, A., Amaratunga, C., Amato, R., Amenga-Etego, L., Andagalu, B., Anderson, T.J.C., et al. (2021). An open dataset of Plasmodium falciparum genome variation in 7,000 worldwide samples. Wellcome Open Res. *6*, 42. https://doi.org/10.12688/wellcomeopenres.16168.2.

26. Regep, C., Georges, G., Shi, J., Popovic, B., and Deane, C.M. (2017). The H3 loop of antibodies shows unique structural characteristics. Proteins *85*, 1311–1318. https://doi.org/10.1002/prot.25291.

27. Baek, M., DiMaio, F., Anishchenko, I., Dauparas, J., Ovchinnikov, S., Lee, G.R., Wang, J., Cong, Q., Kinch, L.N., Schaeffer, R.D., et al. (2021). Accurate prediction of protein structures and interactions using a three-track neural network. Science *373*, 871–876. https://doi.org/10.1126/science.abj8754.

28. Jumper, J., Evans, R., Pritzel, A., Green, T., Figurnov, M., Ronneberger, O., Tunyasuvunakool, K., Bates, R., Žídek, A., Potapenko, A., et al. (2021). Highly accurate protein structure prediction with AlphaFold. Nature *596*, 583–589. https://doi.org/10.1038/s41586-021-03819-2.

29. Yang, J., Yan, R., Roy, A., Xu, D., Poisson, J., and Zhang, Y. (2015). The I-TASSER Suite: protein structure and function prediction. Nat. Methods *12*, 7–8. https://doi.org/10.1038/nmeth.3213.

30. Xu, J., Mcpartlon, M., and Li, J. (2021). Improved protein structure prediction by deep learning irrespective of co-evolution information. Nat. Mach. Intell. *3*, 601–609. https://doi.org/10.1038/s42256-021-00348-5.

31. Kumar, A., Planchais, C., Fronzes, R., Mouquet, H., and Reyes, N. (2020). Binding mechanisms of therapeutic antibodies to human CD20. Science *369*, 793–799. https://doi.org/10.1126/science.abb8008.

32. Rougé, L., Chiang, N., Steffek, M., Kugel, C., Croll, T.I., Tam, C., Estevez, A., Arthur, C.P., Koth, C.M., Ciferri, C., et al. (2020). Structure of CD20 in complex with the therapeutic monoclonal antibody rituximab. Science *367*, 1224–1230. https://doi.org/10.1126/science.aaz9356.

33. McLellan, J.S., Chen, M., Joyce, M.G., Sastry, M., Stewart-Jones, G.B.E., Yang, Y., Zhang, B., Chen, L., Srivatsan, S., Zheng, A., et al. (2013). Structure-based design of a fusion glycoprotein vaccine for respiratory syncytial virus. Science *342*, 592–598. https://doi.org/10.1126/science.1243283.

34. Pallesen, J., Wang, N., Corbett, K.S., Wrapp, D., Kirchdoerfer, R.N., Turner, H.L., Cottrell, C.A., Becker, M.M., Wang, L., Shi, W., et al. (2017). Immunogenicity and structures of a rationally designed prefusion MERS-CoV spike antigen. Proc. Natl. Acad. Sci. USA *114*, E7348–E7357. https://doi.org/10.1073/pnas.1707304114.

35. Wrapp, D., Wang, N., Corbett, K.S., Goldsmith, J.A., Hsieh, C.L., Abiona, O., Graham, B.S., and McLellan, J.S. (2020). Cryo-EM structure of the 2019-nCoV spike in the prefusion conformation. Science *367*, 1260–1263. https://doi.org/10.1126/science.abb2507.

36. Julien, J.P., Lee, J.H., Cupo, A., Murin, C.D., Derking, R., Hoffenberg, S., Caulfield, M.J., King, C.R., Marozsan, A.J., Klasse, P.J., et al. (2013). Asymmetric recognition of the HIV-1 trimer by broadly neutralizing antibody PG9. Proc. Natl. Acad. Sci. USA *110*, 4351–4356. https://doi.org/10.1073/pnas.1217537110.

37. McLellan, J.S., Pancera, M., Carrico, C., Gorman, J., Julien, J.P., Khayat, R., Louder, R., Pejchal, R., Sastry, M., Dai, K., et al. (2011). Structure of HIV-1 gp120 V1/V2 domain with broadly neutralizing antibody PG9. Nature *480*, 336–343. https://doi.org/10.1038/nature10696.

38. Zhou, T., Georgiev, I., Wu, X., Yang, Z.Y., Dai, K., Finzi, A., Kwon, Y.D., Scheid, J.F., Shi, W., Xu, L., et al. (2010). Structural basis for broad and potent neutralization of HIV-1 by antibody VRC01. Science *329*, 811–817. https://doi.org/10.1126/science.1192819.

39. Scheid, J.F., Mouquet, H., Ueberheide, B., Diskin, R., Klein, F., Oliveira, T.Y.K., Pietzsch, J., Fenyo, D., Abadir, A., Velinzon, K., et al. (2011). Sequence and structural convergence of broad and potent HIV antibodies that mimic CD4 binding. Science *333*, 1633–1637. https://doi.org/10.1126/science.1207227.

40. Blattner, C., Lee, J.H., Sliepen, K., Derking, R., Falkowska, E., de la Peña, A.T., Cupo, A., Julien, J.P., van Gils, M., Lee, P.S., et al. (2014). Structural delineation of a quaternary, cleavage-dependent epitope at the gp41-gp120 interface on intact HIV-1 Env trimers. Immunity *40*, 669–680. https://doi.org/10.1016/j.immuni.2014.04.008.

41. Kong, L., Lee, J.H., Doores, K.J., Murin, C.D., Julien, J.P., McBride, R., Liu, Y., Marozsan, A., Cupo, A., Klasse, P.J., et al. (2013). Supersite of immune vulnerability on the glycosylated face of HIV-1 envelope glycoprotein gp120. Nat. Struct. Mol. Biol. *20*, 796–803. https://doi.org/10.1038/nsmb.2594.

42. Moore, P.L., Gray, E.S., Wibmer, C.K., Bhiman, J.N., Nonyane, M., Sheward, D.J., Hermanus, T., Bajimaya, S., Tumba, N.L., Abrahams, M.R., et al. (2012). Evolution of an HIV glycan-dependent broadly neutralizing antibody epitope through immune escape. Nat. Med. *18*, 1688–1692. https://doi.org/10.1038/nm.2985.

43. Sanders, R.W., van Gils, M.J., Derking, R., Sok, D., Ketas, T.J., Burger, J.A., Ozorowski, G., Cupo, A., Simonich, C., Goo, L., et al. (2015). HIV-1 vaccines. HIV-1 neutralizing antibodies induced by native-like envelope trimers. Science *349*, aac4223. https://doi.org/10.1126/science.aac4223.

44. Turner, H.L., Andrabi, R., Cottrell, C.A., Richey, S.T., Song, G., Callaghan, S., Anzanello, F., Moyer, T.J., Abraham, W., Melo, M., et al. (2021). Disassembly of HIV envelope glycoprotein trimer immunogens is driven by antibodies elicited via immunization. Sci. Adv. *7*, eabh2791. https://doi.org/10.1126/sciadv.abh2791.

45. Cheng, C., Pancera, M., Bossert, A., Schmidt, S.D., Chen, R.E., Chen, X., Druz, A., Narpala, S., Doria-Rose, N.A., McDermott, A.B., et al. (2015). Immunogenicity of a prefusion HIV-1 envelope trimer in complex with a quaternary-structure-specific antibody. J. Virol. *90*, 2740–2755. https://doi.org/10.1128/JVI.02380-15.

46. Pauthner, M., Havenar-Daughton, C., Sok, D., Nkolola, J.P., Bastidas, R., Boopathy, A.V., Carnathan, D.G., Chandrashekar, A., Cirelli, K.M., Cottrell, C.A., et al. (2017). Elicitation of robust tier 2 neutralizing antibody responses in nonhuman primates by HIV envelope trimer immunization using optimized approaches. Immunity *46*, 1073–1088.e6. https://doi.org/10.1016/j.immuni.2017.05.007.

47. Bianchi, M., Turner, H.L., Nogal, B., Cottrell, C.A., Oyen, D., Pauthner, M., Bastidas, R., Nedellec, R., McCoy, L.E., Wilson, I.A., et al. (2018). Electron-microscopy-based epitope mapping defines specificities of polyclonal antibodies elicited during HIV-1 BG505 envelope trimer immunization. Immunity *49*, 288–300.e8. https://doi.org/10.1016/j.immuni.2018.07.009.

48. Kulp, D.W., Steichen, J.M., Pauthner, M., Hu, X., Schiffner, T., Liguori, A., Cottrell, C.A., Havenar-Daughton, C., Ozorowski, G., Georgeson, E., et al. (2017). Structure-based design of native-like HIV-1 envelope trimers to silence non-neutralizing epitopes and eliminate CD4 binding. Nat. Commun. *8*, 1655. https://doi.org/10.1038/s41467-017-01549-6.

49. Ringe, R.P., Ozorowski, G., Rantalainen, K., Struwe, W.B., Matthews, K., Torres, J.L., Yasmeen, A., Cottrell, C.A., Ketas, T.J., LaBranche, C.C., et al. (2017). Reducing V3 antigenicity and immunogenicity on soluble, native-like HIV-1 env SOSIP trimers. J. Virol. *91*, e00677-17. https://doi.org/10.1128/JVI.00677-17.

50. Hessell, A.J., Powell, R., Jiang, X., Luo, C., Weiss, S., Dussupt, V., Itri, V., Fox, A., Shapiro, M.B., Pandey, S., et al. (2019). Multimeric epitope-scaffold HIV vaccines target V1V2 and differentially tune polyfunctional antibody responses. Cell Rep. *28*, 877–895.e6. https://doi.org/10.1016/j.celrep.2019.06.074.

51. Eggink, D., Goff, P.H., and Palese, P. (2014). Guiding the immune response against influenza virus hemagglutinin toward the conserved

stalk domain by hyperglycosylation of the globular head domain. J. Virol. *88*, 699–704. https://doi.org/10.1128/JVI.02608-13.

52. Sesterhenn, F., Yang, C., Bonet, J., Cramer, J.T., Wen, X., Wang, Y., Chiang, C.I., Abriata, L.A., Kucharska, I., Castoro, G., et al. (2020). De novo protein design enables the precise induction of RSV-neutralizing antibodies. Science *368*, eaay5051. https://doi.org/10.1126/science.aay5051.

53. Correia, B.E., Bates, J.T., Loomis, R.J., Baneyx, G., Carrico, C., Jardine, J.G., Rupert, P., Correnti, C., Kalyuzhniy, O., Vittal, V., et al. (2014). Proof of principle for epitope-focused vaccine design. Nature *507*, 201–206. https://doi.org/10.1038/nature12966.

54. Scaria, P.V., Anderson, C., Muratova, O., Alani, N., Trinh, H.V., Nadakal, S.T., Zaidi, I., Lambert, L., Beck, Z., Barnafo, E.K., et al. (2021). Malaria transmission-blocking conjugate vaccine in ALFQ adjuvant induces durable functional immune responses in rhesus macaques. npj Vaccines *6*, 148. https://doi.org/10.1038/s41541-021-00407-3.

55. Kaslow, D.C., Quakyi, I.A., Syin, C., Raum, M.G., Keister, D.B., Coligan, J.E., McCutchan, T.F., and Miller, L.H. (1988). A vaccine candidate from the sexual stage of human malaria that contains EGF-like domains. Nature *333*, 74–76. https://doi.org/10.1038/333074a0.

56. Broni, F.K., Acquah, F.K., Obiri-Yeboah, D., Obboh, E.K., Sarpong, E., and Amoah, L.E. (2021). Profiling the quality and quantity of naturally induced antibody responses against Pfs230 and Pfs48/45 among non-febrile children living in Southern Ghana: a longitudinal study. Front. Cell. Infect. Microbiol. *11*, 770821. https://doi.org/10.3389/fcimb.2021.770821.

57. Ayanful-Torgby, R., Sarpong, E., Abagna, H.B., Donu, D., Obboh, E., Mensah, B.A., Adjah, J., Williamson, K.C., and Amoah, L.E. (2021). Persistent Plasmodium falciparum infections enhance transmission-reducing immunity development. Sci. Rep. *11*, 21380. https://doi.org/10.1038/s41598-021-00973-5.

58. Kundu, P., Semesi, A., Jore, M.M., Morin, M.J., Price, V.L., Liang, A., Li, J., Miura, K., Sauerwein, R.W., King, C.R., et al. (2018). Structural delineation of potent transmission-blocking epitope I on malaria antigen Pfs48/45. Nat. Commun. *9*, 4458. https://doi.org/10.1038/s41467-018-06742-9.

59. McLeod, B., Miura, K., Scally, S.W., Bosch, A., Nguyen, N., Shin, H., Kim, D., Volkmuth, W., Rämisch, S., Chichester, J.A., et al. (2019). Potent antibody lineage against malaria transmission elicited by human vaccination with Pfs25. Nat. Commun. *10*, 4328. https://doi.org/10.1038/s41467-019-11980-6.

60. Pholcharee, T., Oyen, D., Flores-Garcia, Y., Gonzalez-Paez, G., Han, Z., Williams, K.L., Volkmuth, W., Emerling, D., Locke, E., Richter King, C., et al. (2021). Structural and biophysical correlation of anti-NANP antibodies with in vivo protection against P. falciparum. Nat. Commun. *12*, 1063. https://doi.org/10.1038/s41467-021-21221-4.

61. Singh, S.K., Thrane, S., Chourasia, B.K., Teelen, K., Graumans, W., Stoter, R., van Gemert, G.J., van de Vegte-Bolmer, M.G., Nielsen, M.A., Salanti, A., et al. (2019). Pfs230 and Pfs48/45 fusion proteins elicit strong transmission-blocking antibody responses against Plasmodium falciparum. Front. Immunol. *10*, 1256. https://doi.org/10.3389/fimmu.2019.01256.

62. Ponnudurai, T., Lensen, A.H., Van Gemert, G.J., Bensink, M.P., Bolmer, M., and Meuwissen, J.H. (1989). Infectivity of cultured Plasmodium falciparum gametocytes to mosquitoes. Parasitology *98*, 165–173. https://doi.org/10.1017/s0031182000062065.

63. Adams, P.D., Afonine, P.V., Bunkóczi, G., Chen, V.B., Davis, I.W., Echols, N., Headd, J.J., Hung, L.W., Kapral, G.J., Grosse-Kunstleve, R.W., et al. (2010). Phenix: a comprehensive Python-based system for macromolecular structure solution. Acta Crystallogr. D Biol. Crystallogr. *66*, 213–221. https://doi.org/10.1107/S0907444909052925.

64. Evans, P.R., and Murshudov, G.N. (2013). How good are my data and what is the resolution? Acta Crystallogr. D Biol. Crystallogr. *69*, 1204–1214. https://doi.org/10.1107/S0907444913000061.

65. Kabsch, W. (2010). XDS. Acta Crystallogr. D Biol. Crystallogr. *66*, 125–132. https://doi.org/10.1107/S0907444909047337.

66. Emsley, P., Lohkamp, B., Scott, W.G., and Cowtan, K. (2010). Features and development of coot. Acta Crystallogr. D Biol. Crystallogr. *66*, 486–501. https://doi.org/10.1107/S0907444910007493.

67. Li, H. (2011). A statistical framework for SNP calling, mutation discovery, association mapping and population genetical parameter estimation from sequencing data. Bioinformatics *27*, 2987–2993. https://doi.org/10.1093/bioinformatics/btr509.

68. Jones, B.E., Brown-Augsburger, P.L., Corbett, K.S., Westendorf, K., Davies, J., Cujec, T.P., Wiethoff, C.M., Blackbourne, J.L., Heinz, B.A., Foster, D., et al. (2021). The neutralizing antibody, LY-CoV555, protects against SARS-CoV-2 infection in nonhuman primates. Sci. Transl. Med. *13*, eabf1906. https://doi.org/10.1126/scitranslmed.abf1906.

69. Vermeulen, A.N., Ponnudurai, T., Beckers, P.J., Verhave, J.P., Smits, M.A., and Meuwissen, J.H. (1985). Sequential expression of antigens on sexual stages of Plasmodium falciparum accessible to transmission-blocking antibodies in the mosquito. J. Exp. Med. *162*, 1460–1476. https://doi.org/10.1084/jem.162.5.1460.

70. Vos, M.W., Stone, W.J.R., Koolen, K.M., van Gemert, G.J., van Schaijk, B., Leroy, D., Sauerwein, R.W., Bousema, T., and Dechering, K.J. (2015). A semi-automated luminescence based standard membrane feeding assay identifies novel small molecules that inhibit transmission of malaria parasites by mosquitoes. Sci. Rep. *5*, 18704. https://doi.org/10.1038/srep18704.

71. Lee, S.M., Wu, Y., Hickey, J.M., Miura, K., Whitaker, N., Joshi, S.B., Volkin, D.B., Richter King, C., and Plieskatt, J. (2019). The Pfs230 N-terminal fragment, Pfs230D1+: expression and characterization of a potential malaria transmission-blocking vaccine candidate. Malar. J. *18*, 356. https://doi.org/10.1186/s12936-019-2989-2.

72. McCoy, A.J., Grosse-Kunstleve, R.W., Adams, P.D., Winn, M.D., Storoni, L.C., and Read, R.J. (2007). Phaser crystallographic software. J. Appl. Crystallogr. *40*, 658–674. https://doi.org/10.1107/S0021889807021206.

73. Morin, A., Eisenbraun, B., Key, J., Sanschagrin, P.C., Timony, M.A., Ottaviano, M., and Sliz, P. (2013). Collaboration gets the most out of software. eLife *2*, e01456. https://doi.org/10.7554/eLife.01456.

74. McLeod, B., Mabrouk, M.T., Miura, K., Ravichandran, R., Kephart, S., Hailemariam, S., Pham, T.P., Semesi, A., Kucharska, I., Kundu, P., et al. (2022). Vaccination with a structure-based stabilized version of malarial antigen Pfs48/45 elicits ultra-potent transmission-blocking antibody responses. Immunity *55*, 1680–1692.e8. https://doi.org/10.1016/j.immuni.2022.07.015.

75. de Jong, R.M., Singh, S.K., Teelen, K., van de Vegte-Bolmer, M., van Gemert, G.J., Stone, W.J.R., Locke, E., Plieskatt, J., Theisen, M., Bousema, T., et al. (2022). Heterologous expression and evaluation of novel Plasmodium falciparum transmission blocking vaccine candidates. Front. Immunol. *13*, 909060. https://doi.org/10.3389/fimmu.2022.909060.

76. van der Boor, S.C., Smit, M.J., van Beek, S.W., Ramjith, J., Teelen, K., van de Vegte-Bolmer, M., van Gemert, G.J., Pickkers, P., Wu, Y., Locke, E., et al. (2022). Safety, tolerability, and Plasmodium falciparum transmission-reducing activity of monoclonal antibody TB31F: a single-centre, open-label, first-in-human, dose-escalation, phase 1 trial in healthy malaria-naive adults. Lancet Infect. Dis. *22*, 1596–1605. https://doi.org/10.1016/S1473-3099(22)00428-5.

STAR★METHODS

KEY RESOURCES TABLE

REAGENT or RESOURCE	SOURCE	IDENTIFIER
**Antibodies**		
RUPA-32 (IgG1)	This paper	N/A
RUPA-33 (IgG1)	This paper	N/A
RUPA-38 (IgG1)	This paper	N/A
RUPA-39 (IgG1)	This paper	N/A
RUPA-55 (IgG1)	This paper	N/A
RUPA-62 (IgG1)	This paper	N/A
RUPA-73 (IgG1)	This paper	N/A
RUPA-96 (IgG1)	This paper	N/A
RUPA-97 (IgG1)	This paper	N/A
RUPA-103 (IgG1)	This paper	N/A
RUPA-115 (IgG1)	This paper	N/A
RUPA-126 (IgG1)	This paper	N/A
RUPA-133 (IgG1)	This paper	N/A
RUPA-135 (IgG1)	This paper	N/A
RUPA-136 (IgG1)	This paper	N/A
RUPA-137 (IgG1)	This paper	N/A
LMIV230-01 (IgG1)	Coelho et al.[18]	N/A
LMIV230-02 (IgG1)	Coelho et al.[18]	N/A
15C5 (IgG1)	Lee et al.[23]	N/A
hu4F12 (IgG1)	MacDonald et al.[14]	N/A
TB31F (IgG1)	Kundu et al.[58]	N/A
hu2A2 (IgG1)	This paper	N/A
RUPA-32 (Fab)	This paper	N/A
RUPA-38 (Fab)	This paper	N/A
RUPA-55 (Fab)	This paper	N/A
RUPA-97 (Fab)	This paper	N/A
LMIV230-01 (Fab)	Coelho et al.[18]	N/A
LMIV230-02 (Fab)	Coelho et al.[18]	N/A
15C5 (Fab)	Lee et al.[23]	N/A
hu4F12 (Fab)	MacDonald et al.[14]	N/A
Alexa Fluor® 488 AffiniPure $F(ab')_2$ fragment goat anti-human IgG, Fcγ fragment specific	Jackson Laboratories	Cat#109-546-098
AffiniPure Rabbit Anti-Human IgG, Fcγ fragment specific	Jackson Laboratories	Cat#309-005-008
2544 (IgG1)	McLeod et al.[59]	N/A
399 (IgG1)	Pholcharee et al.[60]	N/A
anti-Human IgG-HRP	ThermoFisher	Cat# 31412
anti-human IgG-AF488	Invitrogen	Cat# A-11013
**Biological samples**		
PBMCs and plasma from Ugandan donor	Kamya et al.[22]	N/A
PBMCs and plasma from Dutch expatriate	Stone et al.[21]	N/A
**Chemicals, peptides, and recombinant proteins**		
Pfs230 C1	Lee et al.[23]	N/A
Pfs230 Pro	Singh et al.[61]	N/A

(*Continued on next page*)

Continued

REAGENT or RESOURCE	SOURCE	IDENTIFIER
Pfs230 D1+ (C terminal 6x STREP II tag)	This paper	N/A
Pfs230 D1+ (C terminal 6x HIS tag)	This paper	N/A
Pfs230 D1+ (N719S; C terminal 6x HIS tag)	This paper	N/A
Pfs230 D1+ (K716N; C terminal 6x HIS tag)	This paper	N/A
Pfs230 D1+ (V632A; C terminal 6x HIS tag)	This paper	N/A
Pfs230 D1+ (E612K; C terminal 6x HIS tag)	This paper	N/A
Pfs230 D1+ (T602K; C terminal 6x HIS tag)	This paper	N/A
Pfs230 D1+ (A583T; C terminal 6x HIS tag)	This paper	N/A
GIBCO™ FreeStyle™ 293 Expression Medium	Thermo Fisher Scientific	Cat#12338026
FectoPRO DNA Transfection Reagent	VWR	Cat#10118-444
DyLight™ 488 NHS Ester	Thermo Fisher	Cat#46403
DyLight™ 650 NHS Ester	Thermo Fisher	Cat#62265
Pierce IgG elution buffer	Thermo Fisher	Cat#21004
10X HBSTE running buffer	Carterra	Cat#3630
0.1 M MES	Carterra	Cat#3625
10 mM sodium acetate buffer	Carterra	Cat#3801
10 mM glycine pH 2.0	Carterra	Cat#3640
Sulfo–N-hydroxysuccinimide (sulfo-NHS)	Thermo Fisher	Cat#24510
1-ethyl-3-(3-dimethylaminopropyl) carbodiimide (EDC)	Thermo Fisher	Cat#22980
**Critical commercial assays**		
Ni-NTA biosensors	ForteBio	Cat#18-5102
Anti-human Fab-CH1 biosensors	ForteBio	Cat#18-5125
HC30M chip	Carterra	Cat#4279
**Deposited data**		
Crystal structure of Pfs230 domain 1 bound by RUPA-32 Fab	This paper	PDB: 7UVH
Crystal structure of Pfs230 domain 1 bound by RUPA-38 Fab	This paper	PDB: 7UVO
Crystal structure of Pfs230 domain 1 bound by RUPA-55 Fab	This paper	PDB: 7UVI
Crystal structure of Pfs230 domain 1 bound by RUPA-97 and 15C5 Fabs	This paper	PDB: 7UVQ
Crystal structure of Pfs230 domain 1 bound by LMIV230-02 Fab	This paper	PDB: 7UVS
**Experimental models: Cell lines**		
FreeStyle™ 293-F Cells	Thermo Fisher Scientific	Cat#R79007
**Experimental models: Organisms/strains**		
Parasite: *P. falciparum ; NF54 strain*	Ponnudurai et al.[62]	N/A
Mosquito: *Anopheles stephensi* (Nijmegen strain)	Ponnudurai et al.[62]	N/A
**Recombinant DNA**		
pcDNA3.4_RUPA-32 (Fab)	This paper	N/A
pcDNA3.4_RUPA-38 (Fab)	This paper	N/A
pcDNA3.4_RUPA-55 (Fab)	This paper	N/A
pcDNA3.4_RUPA-97 (Fab)	This paper	N/A
pcDNA3.4_LMIV230-01 (Fab)	Coelho et al.[18]	N/A
pcDNA3.4_LMIV230-02 (Fab)	Coelho et al.[18]	N/A

(*Continued on next page*)

Continued

REAGENT or RESOURCE	SOURCE	IDENTIFIER
pcDNA3.4_15C5 (Fab)	Lee et al.[23]	N/A
pcDNA3.4_hu4F12 (Fab)	MacDonald et al.[14]	N/A
pcDNA3.4_RUPA-32 (Fab)	This paper	N/A
pcDNA3.4_RUPA-38 (Fab)	This paper	N/A
pcDNA3.4_RUPA-55 (Fab)	This paper	N/A
pcDNA3.4_Pfs230 D1+ (C terminal 6x STREP II tag)	This paper	N/A
pcDNA3.4_Pfs230 D1+ (C terminal 6x HIS tag)	This paper	N/A
pcDNA3.4_Pfs230 D1+ (N719S; C terminal 6x HIS tag)	This paper	N/A
pcDNA3.4_Pfs230 D1+ (K716N; C terminal 6x HIS tag)	This paper	N/A
pcDNA3.4_Pfs230 D1+ (V632A; C terminal 6x HIS tag)	This paper	N/A
pcDNA3.4_Pfs230 D1+ (E612K; C terminal 6x HIS tag)	This paper	N/A
pcDNA3.4_Pfs230 D1+ (T602K; C terminal 6x HIS tag)	This paper	N/A
pcDNA3.4_Pfs230 D1+ (A583T; C terminal 6x HIS tag)	This paper	N/A
**Software and algorithms**		
PRISM Graphpad	GraphPad Software, LLC	https://www.graphpad.com/scientific-software/prism/
Phenix	Adams et al.[63]	http://www.phenix-online.org/
Aimless	Evans et al.[64]	https://www.mrc-lmb.cam.ac.uk/harry/pre/aimless.html
XDS	Kabsch et al.[65]	https://xds.mr.mpg.de/html_doc/downloading.html
Coot	Emsley et al.[66]	https://www2.mrc-lmb.cam.ac.uk/personal/pemsley/coot/
PyMOL	Schrödinger, LLC.	The PyMOL Molecular Graphics System, v2.3.4.
Bcftools	Li et al.[67]	https://samtools.github.io/bcftools/bcftools.html
RoseTTAFold	Baek et al.[27]	https://robetta.bakerlab.org/collabfor
AlphaFold2	Jumper et al.[28]	https://colab.research.google.com/github/sokrypton/ColabFold/blob/main/AlphaFold2.ipynbl
I-Tasser	Yang et al.[29]	https://zhanggroup.org/I-TASSER/
RaptorX	Xu et al.[30]	http://raptorx.uchicago.edu/
Kinetics analysis software	Carterra	https://carterra-bio.com/applications/kinetics-software/
Epitope analysis software	Carterra	https://carterra-bio.com/applications/epitope-binning-software
MetaXpress	Molecular Devices	https://www.moleculardevices.com/products/cellular-imaging-systems/acquisition-and-analysis-software/metaxpress
R (version 4.1.2)	The R Foundation	https://www.r-project.org/foundation/
**Other**		
QuantumPlex^{TM} optically encoded beads	Bangs Laboratories	Cat#235
Polystyrene beads	Bangs Laboratories	Cat#PC06N

RESOURCE AVAILABILITY

Lead Contact

Further information and requests for resources and reagents should be directed to and will be fulfilled by the Lead Contact, Jean-Philippe Julien (jean-philippe.julien@sickkids.ca).

Materials Availability

All unique reagents generated in this study are available via the lead contact upon a request.

Data and Code Availability

- Antibody sequences are available in Table S1. Crystal structures have been deposited in the Protein Data Bank and are publicly available as of the date of publication. PDB IDs are listed in the key resources table.
- This paper does not report original code.
- Any additional information required to reanalyze the data reported in this paper is available from the lead contact upon request.

EXPERIMENTAL MODELS AND SUBJECT DETAILS

Donor selection and PBMC collection

Plasma samples and peripheral blood mononuclear cells (PBMCs) were collected from a 69-year-old male Dutch expatriate and from volunteers enrolled in the East African International Centers of Excellence in Malaria Research "PRISM" Tororo study cohort in Tororo, Uganda between 2013 and 2017; at enrolment 46% of participants were female, the age range was 6 months-68 years and none were symptomatic.[22] PBMCs from the Dutch expatriate were isolated in 1994, using a Percoll gradient. For the selected Ugandan donor, an asymptomatic 8-year-old female who at the moment of phlebotomy had an uncomplicated malaria infection with self-reported fever and a *P. falciparum* parasite density of 2080 parasites/μl, PBMCs were isolated from blood collected in acid citrate dextrose (ACD) tubes by Ficoll gradient. Written informed consent was obtained from the parent/guardian of the study participant, and study protocols were approved by the Uganda National Council of Science and Technology (HS 1019), the Makerere University School of Medicine Research and Ethics Committee (Rec No. 2011–167), and the University of California, San Francisco Committee on Human Research (11–05995).

Human cell line culture

For expression of recombinant proteins, a female human cell line (HEK 293F, FreeStyle™ 293-F cells, Thermo Fisher Scientific) was cultured in suspension in GIBCO™ FreeStyle™ 293 Expression Medium (Thermo Fisher Scientific) for 6-7 days at 37°C, with 70% humidity and 8% CO_2 and rotating at 150 RPM. The HEK 293F cells were not further authenticated as they were used for routine expression of mAbs and antigens.

METHOD DETAILS

Single B-cell screening and recovery

Single B cell screening and recovery was done as previously described.[68] Briefly, cells were thawed, activated in culture to generate memory B cells, and enriched for antibody secreting cells before injection into AbCellera's microfluidic screening devices with either 91,000 or 153,000 individual nanoliter-volume reaction chambers. Single cells secreting Pfs230-C1-specific antibodies were identified and isolated using two screening assays. In the multiplexed bead assay, multiple optically encoded beads were each conjugated to a different *Plasmodium* antigen. Unique mAbs binding specifically to the antigens on beads were detected by a fluorescently labeled anti-human IgG secondary antibody. In the soluble antigen assay, beads conjugated with anti-human IgG antibody were used to capture secreted IgG via the Fc region (Figure S1). Beads were flowed into microfluidic screening devices, incubated with single antibody-secreting cells, and unique mAbs binding to cognate antigens were detected by soluble *Plasmodium* antigens labeled with fluorophores. Positive hits were identified using machine vision and recovered using automated robotics-based protocols.

Single B-cell sequencing, bioinformatic analysis and antibody expression

Single-cell polymerase chain reaction (PCR) and custom molecular biology protocols generated next-generation sequencing libraries (MiSeq, Illumina) using automated workstations (Bravo, Agilent). Sequencing data were analyzed using a custom bioinformatics pipeline to yield paired heavy and light chain sequences for recovered antibody-secreting cells.[68] Antibody sequences can be found in Table S1. Each sequence was annotated with the closest germline (V(D)J) genes and degree of somatic hypermutation. Antibodies were considered members of the same clonal family if they shared the same inferred heavy and light V and J genes, and had the same CDR3 length. The variable (V(D)J) region of each antibody chain was synthesized and inserted into expression plasmids and produced as recombinant human IgG (GenScript).

Affinity measurements and epitope binning

All high-throughput SPR binding and epitope binning experiments were performed on a Carterra LSA instrument equipped with an HC-30M chip type (Carterra-bio) using a 384-ligand array format as previously described (https://www.biorxiv.org/content/10.1101/2021.04.30.442182v3.full.pdf and[68]). For all experiments, antibodies were coupled to the HC-30M chip: the chip surface was first activated by flowing a freshly prepared 1:1:1 activation mix of 100 mM MES (pH 5.5), 100 mM sulfo–N-hydroxysuccinimide, and 400 mM 1-ethyl-3-(3-dimethylaminopropyl)carbodiimide for 7 min, and antibodies diluted to either 10 or 1 μg/ml in 10 mM NaOAc (pH 4.25) buffer + 0.01% Tween were injected and printed simultaneously onto the chip surface for 10 min by direct coupling. The chip surface was quenched by flowing 1 M EtOHamine for 7 min, followed by two wash steps of 15 s each in 25 mM MES (pH 5.5) buffer. Relevant benchmarks and negative control antibodies were also printed on the chip surface.

For binding kinetics and affinity measurements, a threefold dilution series of the antigen of interest, starting at 500 nM in HEPES-buffered saline containing 0.05% Tween 20 and 3 mM EDTA (HBSTE) + 0.05% BSA running buffer, was sequentially injected onto the chip surface. For each concentration, the antigen was injected for 5 min (association phase), followed by running buffer injection for 15 min (dissociation phase). Two regeneration cycles of 60 s were performed between each dilution series by injecting Pierce IgG elution buffer (Thermo Fisher Scientific) + 1 M NaCl on the chip surface.

High-throughput epitope binning experiments were performed in a classical sandwich assay format. Antibodies were immobilized to the chip at 10 ug/mL as outlined above and antigen at 40 nM was injected for 3 min followed immediately by an antibody analyte at 30 μg/ml for 4 min (both antigen and antibody diluted in 1× HBSTE + 0.05% BSA running buffer). Surfaces were regenerated with 10 mM glycine (pH 2.0) using two 20 s regeneration cycles. An antigen-only injection (40 nM concentration in running buffer) was performed every 8 cycles. The data were analyzed using the Carterra Epitope analysis software for heatmap and competition network generation. Binding responses were normalized to 1 at the end of the antigen binding step. A report time point was set at the end of the antibody analyte step to read out the competition response, relative to the response of the buffer blank analytes at this time point that was nominally set to zero. A threshold was set above this value, such that normalized responses <0.2 were considered blockers and normalized responses >0.3 were considered sandwichers. Normalized responses falling within these limits (0.2-0.3) were considered ambiguous. Antibodies with low coupling to the chip, poor regeneration, or absence of self-blocking were excluded from the binning analysis. Like-behaved antibodies were automatically clustered to form a heatmap and competition plot.

Enzyme-linked immunosorbent assay (ELISA)

Microtiter plates (Nunc MaxiSorp, ThermoFisher) were coated with 1 μg/ml Pfs230-C1 (amino acids 443-731)[23] or Pfs230-Pro (amino acids 443-590)[61] in PBS. Plates were washed with PBS, blocked with 5% milk in PBS for 1 h at room temperature, and washed again. Plates were then incubated with plasma or monoclonal antibodies that were diluted in 1% milk in PBS + 0.05% Tween-20 (PBST), for 3 h at room temperature. Plates were washed and incubated with anti-Human IgG-HRP in PBST (1:60,000 dilution, ThermoFisher, cat no. 31412) for 1 h at room temperature. After a final washing step, BioFX TMB substrate (Surmodics) was added. The reaction was stopped with 0.2 M H_2SO_4 and the optical density was read at 450 nm with an iMark plate reader (Bio-Rad).

Western blot

Plasmodium falciparum NF54 gametocytes were cultured *in vitro* as previously described.[69] Fourteen-day old mature gametocytes were then purified with 2 centrifugation steps of 15 min each, at 1,250 g; in the first step gametocytes were passed through a 40% Percoll + PBS layer, and in the second step the resuspended gametocytes were loaded on a 63% Percoll + PBS layer and after centrifugation purified gametocytes were collected from the Percoll interface. Gametocyte extract was generated by incubating gametocytes with 1% Sodium Deoxycholate + 5 μM PMSF + 20 mM NaCl + 20 mM TrisHCl pH 8.0. Solubilized proteins were separated on a 4-12% SDS PAGE gel (NOVEX), transferred to a nitrocellulose membrane and strips were cut, each strip containing extract of 250,000 gametocytes. Strips were blocked with 5% milk in PBST, washed, incubated with 5 μg/ml monoclonal antibody in PBST, washed, incubated with anti-human IgG-HRP (1:5,000 dilution, Pierce, Cat no. 31412) and washed again. Strips were developed using Clarity Western ECL (BioRad) and imaged with an ImageQuant LAS 4000 system (GE Healthcare).

Surface immunofluorescence assay (SIFA)

In vitro cultured *Plasmodium falciparum* NF54 gametocytes were activated to generate female gametes, which were purified with Nycodenz.[69] Per condition 10,000 female gametes were incubated with monoclonal antibodies diluted in SIFA buffer (1% heat-inactivated FCS, 0.05% sodium azide in PBS) for 1 h at 4°C. Samples were washed 3 times with SIFA buffer. Gametes were stained with Hoechst 33342 DNA stain (1:200 dilution) (Invitrogen, cat no. H3570) and anti-human IgG-AF488 (1:400 dilution) (Invitrogen, cat no. A-11013) for 1 h at 4°C in the dark. Gametes were then washed 3 times with SIFA buffer, fixed with 4% paraformaldehyde and imaged with an ImageXpress Pico automated cell imaging system (Molecular devices). Gametes were then analyzed with MetaXpress software (Molecular devices). Hemozoin and Hoechst-positive gametes were selected and positivity for human antibodies was determined using signal from negative control antibodies as a threshold.

Standard membrane feeding assays (SMFA)

SMFA experiments were conducted as previously described and used either *Plasmodium falciparum* NF54 wildtype gametocytes with oocyst count readout[62] or *P. falciparum* NF54-L1 with oocyst expressed luciferase readout,[70] which can be used interchangeably with assay controls. Purified IgGs from naturally exposed donors were tested with wildtype or luciferase expressing parasites,

depending on availability. Monoclonal antibodies were tested with wild type parasites and oocysts counts only. Blood meals containing cultured *P. falciparum* gametocytes were fed to *Anopheles stephensi* mosquitoes (Nijmegen colony). All SMFA experiments were conducted in the presence of active complement unless stated otherwise, in which case complement was inactivated by incubating at 56°C for 30 min. For each condition 20 fully-fed mosquitoes were analyzed. Reported antibody concentrations are concentrations in the total blood meal volume. Transmission reducing activity was calculated as the reduction in oocysts compared to a negative control. mAb 2A2, which is a complement-dependent mouse antibody that targets Pfs230 Domain 4[10,24] was used to confirm that complement was successfully inactivated.

Fab and antigen expression and purification

Variable light (VL) and heavy (VH) chains of Fabs used in these studies were gene synthesized and cloned (GeneArt) into custom pcDNA3.4 expression vectors immediately upstream of human Igκ and Igγ1-CH1 domains. Fab heavy chain and Fab light chain plasmids were co-transfected at a 2:1 ratio into FreeStyle 293-F cells (Thermo Fisher Scientific) at a cell density of 0.8×10^6 cells/ml for transient expression using FectoPRO DNA transfection reagent (Polyplus). Cells were cultured in GIBCO FreeStyle 293 Expression Medium for 7 days, and supernatants were isolated by centrifugation and filtered through a 0.22-μm membrane. Fabs were purified following a scheme of HiTrap KappaSelect affinity chromatography (Cytiva), cation exchange chromatography (MonoS, Cytiva), and size-exclusion chromatography (Superdex 200 Increase 10/300 GL, Cytiva).

The Pfs230 D1+ amino acid sequence (552–731), containing an N585Q mutation to remove a potential *N*-glycosylation site,[71] was back-translated, codon-optimized for expression in human cells, appended to a C-terminal TEV cleavage site followed by a Strep-tag II sequence, and cloned in a pcDNA3.4 expression vector. Pfs230 D1+ plasmid was transfected into FreeStyle 293-F cells (Thermo Fisher Scientific) at a cell density of 0.8×10^6 cells/ml for transient expression using FectoPRO DNA transfection reagent (Polyplus). Cells were cultured in GIBCO FreeStyle 293 Expression Medium for 7 days, and supernatants were isolated by centrifugation and filtered through a 0.22-μm membrane. Pfs230 D1+ was purified using StrepTrap HP affinity chromatography (Cytiva) and size-exclusion chromatography (Superdex 200 Increase 10/300 GL, Cytiva) into a final buffer of 20 mM Tris and 150 mM sodium chloride at pH 8.0. Purified Pfs230 D1+ protein was concentrated to 1 mg/ml and immediately frozen and stored at -80 °C.

Crystallization and structure determination

Purified Fabs were mixed with Pfs230 D1+ (Strep-tagged) in a 2:1 molar ratio and excess Fab was separated from Fab-antigen complexes using size-exclusion chromatography (Superdex 200 Increase 10/300 GL, Cytiva) in 20 mM Tris and 150 mM sodium chloride at pH 8.0. For the ternary complex of Fab 97 and Fab 15C5 bound to Pfs230 D1+, a 2:2:1 molar ratio of Fab:Fab:antigen was used. Purified Fab 32 and Pfs230 D1+ complex was concentrated to 7.6 mg/ml and mixed in a 1:1 ratio with crystallization buffer (0.2 M ammonium sulfate and 30 % (w/v) PEG 8000). Crystals grew from a sitting-drop, vapour diffusion scheme at 20 °C and were cryoprotected in crystallization buffer supplemented with 15% ethylene glycol, before being flash-frozen in liquid nitrogen. Purified Fab 38 and Pfs230 D1+ complex was concentrated to 5.0 mg/ml and mixed in a 1:1 ratio with crystallization buffer (19% v/v isopropanol, 20% (w/v) PEG 4000, 0.1 M sodium citrate pH 6.5). Crystals grew from a sitting-drop, vapour diffusion scheme at 20 °C and were flash-frozen in liquid nitrogen. Purified Fab 55 and Pfs230 D1+ complex was concentrated to 3.8 mg/ml and mixed in a 1:1 ratio with crystallization buffer (1.2 M sodium dihydrogen phosphate, 0.8 M dipotassium hydrogen phosphate, and 0.2 M lithium sulfate). Crystals grew from a sitting-drop, vapour diffusion scheme at 20 °C and were cryoprotected in crystallization buffer supplemented with 20% ethylene glycol, before being flash-frozen in liquid nitrogen. Purified Fab LMIV230-02 and Pfs230 D1+ complex was concentrated to 9.6 mg/ml and mixed in a 1:1 ratio with crystallization buffer (20 mM dihydrogen phosphate, 20% (w/v) PEG 3350). Crystals grew from a sitting-drop, vapour diffusion scheme at 20 °C and were cryoprotected in crystallization buffer supplemented with 20% ethylene glycol, before being flash-frozen in liquid nitrogen. Purified Fab 97, Fab 15C5 and Pfs230 D1+ complex was concentrated to 8.3 mg/ml and mixed in a 1:1 ratio with crystallization buffer (0.2 M di-ammonium hydrogen citrate, 17.5% (w/v) polyethylene glycol 3350). Crystals grew from a sitting-drop, vapour diffusion scheme at 20 °C and were cryoprotected in crystallization buffer supplemented with 15% ethylene glycol, before being flash-frozen in liquid nitrogen. Data were collected at the 23-ID-D or 23-ID-B beamlines at the Argonne National Laboratory Advanced Photon Source. All datasets were processed and scaled using XDS[65] and Aimless.[64] The structures were determined by molecular replacement using Phaser.[72] Structural refinements were performed using PHENIX[63] and models were manually checked and improved with Coot.[66] Images were generated using PyMOL (The PyMOL Molecular Graphics System, v2.3.4, Schrödinger, LLC.). Access to all software was supported through SBGrid.[73]

Single nucleotide polymorphism detection

Single nucleotide polymorphisms were obtained from the MalariaGEN Catalogue of Genetic Variation in P. falciparum - version 6.0,[25] which contains genotype calls on >6 million SNPs and short indels observed in 7,113 *P. falciparum* samples from 29 countries (73 different locations in Africa, Asia, America, and Oceania). Genotype calls for chromosome 2 (Pf3D7_02_v3) were downloaded (ftp://ngs.sanger.ac.uk/production/malaria/pfcommunityproject/Pf6/Pf_6_vcf/Pf_60_public_Pf3D7_02_v3.final.vcf.gz) and Bcftools[67] was used to subset calls between nucleotide positions 369,351 and 380,156(+) that coincide with Pfs230 and calculate the allele frequency of all polymorphism occurrences. SNPs calls below MalariaGEN's quality filter (Low_VQSLOD) and all silent mutations were removed. The analysis of missense polymorphisms was restricted to the amino acid boundaries of Pfs230 D1+ (552–731) and 30 mutations were identified.

CellPress
OPEN ACCESS

Biolayer interferometry

Direct binding kinetics measurements were conducted at 25 °C using an Octet RED96 instrument (Sartorius ForteBio). To assess the binding kinetics of Fabs for Pfs230 D1+, Fab proteins at 10-20 μg/ml were loaded onto Anti-Human Fab-CH1 2nd Generation (FAB2G) biosensors (Sartorius ForteBio) to reach a BLI signal response of 1.0 nm. Association rates were measured by transferring the loaded biosensors to wells containing Pfs230 D1+ with a C-terminal TEV cleavage site followed by a Strep-tag II in two-fold dilution series from 250 to 16 nM and then dissociation rates were measured by dipping the biosensors carrying Fab-antigen complexes into kinetics buffer (137 mM sodium chloride, 2.7 mM potassium chloride, 10 mM sodium phosphate dibasic dihydrate, and 1.8 mM potassium dihydrogen phosphate, 0.02% Tween-20, and 0.1% BSA). To assess the impact of coding SNP on the binding of antibodies Pfs230D1+, uncleavable C-terminally His-tagged Pfs230D1+ constructs with either the wildtype or single mutant (V632A, T602K, N719S, K716N, E612K, and A583T) sequences were expressed in FreeStyle 293-F cells. Supernatants containing overexpressed proteins were isolated by centrifugation, filtered through a 0.22-μm membrane, and mixed at a 1:1 ratio with kinetics buffer. His-tagged protein from clarified supernatant was loaded onto Ni-NTA (NTA) biosensors (Sartorius ForteBio) to reach a BLI signal response of 1.0 nm. Association rates were measured by transferring the loaded biosensors to wells containing Fab proteins of RUPA-32, 55, 97, or 38, in two-fold dilution series from 125 to 8 nM, and then dissociation rates were measured by dipping the biosensors carrying Fab-antigen complexes into kinetics buffer. Data analysis was performed using the Octet software (Sartorius ForteBio, version 8.2.0.7) and the sensograms were fit using a 1:1 binding model. Binding competition assays were performed using wildtype Pfs230D1+ protein with Fab proteins following a two-step binding sequence in kinetics buffer. C-terminally His-tagged Pfs230D1+ was purified using size exclusion chromatography and loaded onto Ni-NTA (NTA) biosensors (Sartorius ForteBio) at a concentration of 20 μg/ml to reach a BLI signal response of 1.0 nm. Next the Pfs230D1+-loaded biosensors were dipped into wells containing the first antibody (Fab 1) at 50 μg/ml for 300 s and then sensors carrying Fab 1 bound to Pfs230D1+ were dipped into wells containing the second antibody (Fab 2) at 50 μg/ml for an additional 300 s. Data analysis was performed using the Octet software (Sartorius ForteBio, version 8.2.0.7) and the binding competition was quantified as percent ratio of each antibody's BLI signal at each stage of the two-step binding sequence (i.e. Fab2/Fab1 measured in nm).

In silico structure modeling

Theoretical models of Pfs230 domain 1 and 2 (residues 552-889) were generated using RoseTTAFold,[27] AlphaFold2,[28] I-Tasser,[29] and RaptorX.[30] The RoseTTAFold, I-Tasser, and RaptorX platforms, were accessed from their respective publicly available online servers, while a publicly available, accelerated implementation of AlphaFold2 was accessed from the Colabfold server using default parameters (https://www.biorxiv.org/content/10.1101/2021.08.15.456425v2.full). All five output models from each method were visually inspected in PyMOL, while only the highest-ranking output model was selected for analysis with Fabs 15C5 and LMIV230-02. All domain 1 theoretical models structurally overlapped with the experimentally derived Pfs230 domain 1, while both domains 1 and 2 possessed expected disulfide bridges indicating a high confidence in the theoretical model predictions. The position of domain 2 relative to domain 1 was consistent across all models indicating further confidence in the predictions.

QUANTIFICATION AND STATISTICAL ANALYSIS

TRA values were calculated using a negative binomial regression model for samples that were tested in a single SMFA experiment and using a mixed-effects negative binomial regression model for samples that were tested in multiple independent SMFA experiments.[74,75] IC_{80} values were calculated by linear regression analysis on the $\log_{10}$ transformed ratio of mean oocyst count in control and test sample, and square root of antibody concentration, and the 95% confidence intervals for the IC_{80} values were calculated with the delta method.[76] SMFA data analyses were done in R (version 4.1.2).

For binding kinetics measured by surface plasmon resonance, data was analyzed using the Carterra Kinetics analysis software using a 1:1 Langmuir binding model to determine apparent association (k_a) and dissociation (k_d) kinetic rate constants and apparent binding affinity constants (K_D). For binding kinetics calculated by biolayer interferometry, data was analyzed using the Octet software (Sartorius ForteBio, version 8.2.0.7) according to the manufacturer's instructions.

Immunity, Volume *56*

Supplemental information

Potent transmission-blocking monoclonal antibodies from naturally exposed individuals target a conserved epitope on *Plasmodium falciparum* Pfs230

Danton Ivanochko, Amanda Fabra-García, Karina Teelen, Marga van de Vegte-Bolmer, Geert-Jan van Gemert, Jocelyn Newton, Anthony Semesi, Marloes de Bruijni, Judith Bolscher, Jordache Ramjith, Marta Szabat, Stefanie Vogt, Lucas Kraft, Sherie Duncan, Shwu-Maan Lee, Moses R. Kamya, Margaret E. Feeney, Prasanna Jagannathan, Bryan Greenhouse, Robert W. Sauerwein, C. Richter King, Randall S. MacGill, Teun Bousema, Matthijs M. Jore, and Jean-Philippe Julien

Supplementary Figures and Tables

Potent transmission-blocking monoclonal antibodies from naturally exposed individuals target a conserved epitope on *Plasmodium falciparum* Pfs230

Danton Ivanochko[1*], Amanda Fabra-García[2*], Karina Teelen[2], Marga van de Vegte-Bolmer[2], Geert-Jan van Gemert[2], Jocelyn Newton[1], Anthony Semesi[1], Marloes de Bruijni[3], Judith Bolscher[3], Jordache Ramjith[4], Marta Szabat[5], Stefanie Vogt[5], Lucas Kraft[5], Sherie Duncan[5], Shwu-Maan Lee[6], Moses R Kamya[7], Margaret E Feeney[8,9], Prasanna Jagannathan[10], Bryan Greenhouse[8], Robert W Sauerwein[3], C Richter King[6], Randall S MacGill[6], Teun Bousema[2,#], Matthijs M Jore[2,#], Jean-Philippe Julien[1,11,#]

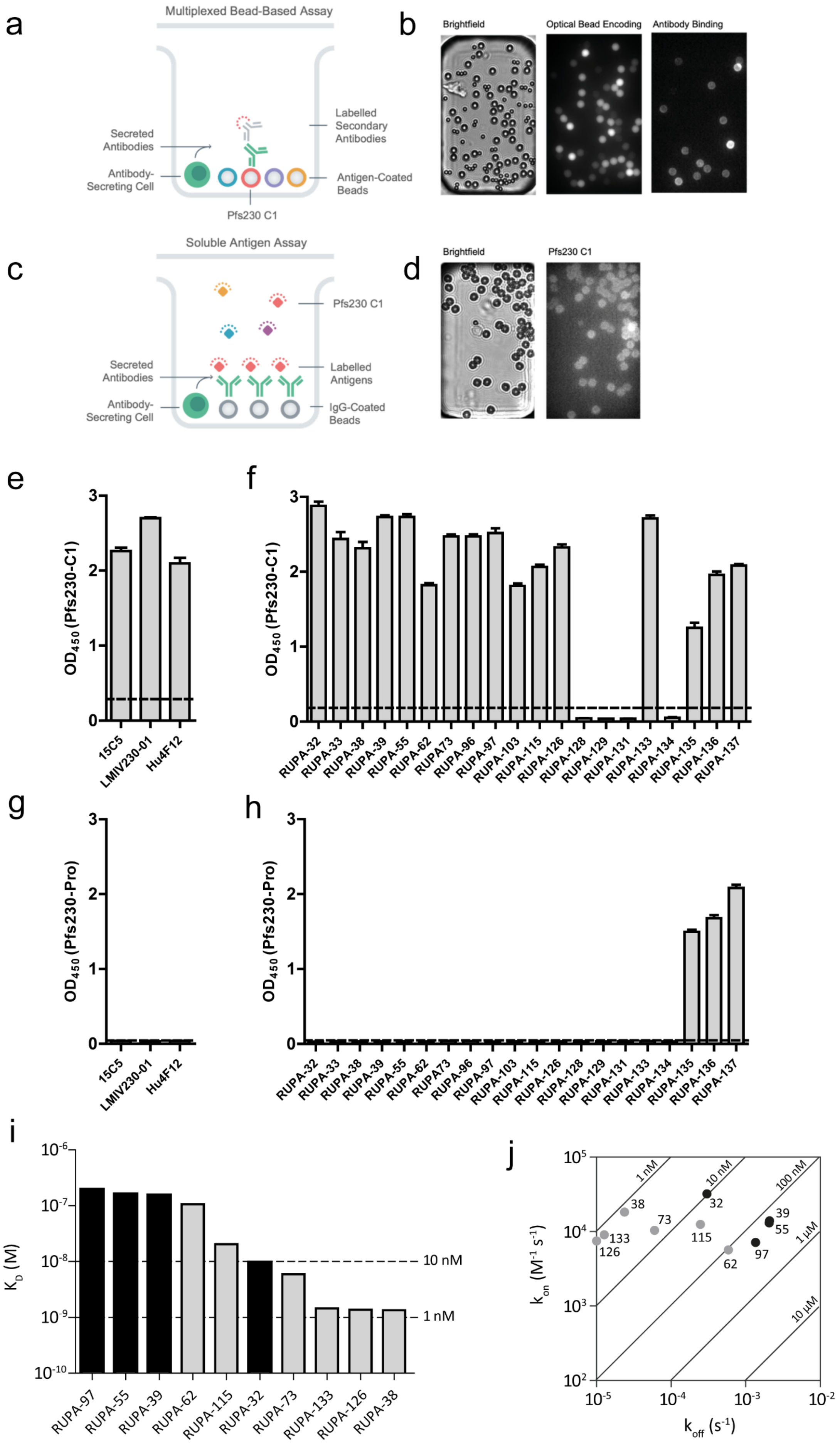
a
Multiplexed Bead-Based Assay
Secreted Antibodies
Antibody-Secreting Cell
Labelled Secondary Antibodies
Antigen-Coated Beads
Pfs230 C1
b
Brightfield
Optical Bead Encoding
Antibody Binding
c
Soluble Antigen Assay
Pfs230 C1
Secreted Antibodies
Labelled Antigens
Antibody-Secreting Cell
IgG-Coated Beads
d
Brightfield
Pfs230 C1
e
OD450 (Pfs230-C1)
15C5
LMIV230-01
Hu4F12
f
RUPA-32 RUPA-33 RUPA-38 RUPA-39 RUPA-55 RUPA-62 RUPA73 RUPA-96 RUPA-97 RUPA-103 RUPA-115 RUPA-126 RUPA-128 RUPA-129 RUPA-131 RUPA-133 RUPA-134 RUPA-135 RUPA-136 RUPA-137
g
OD450 (Pfs230-Pro)
h
i
KD (M)
10 nM
1 nM
RUPA-97 RUPA-55 RUPA-39 RUPA-62 RUPA-115 RUPA-32 RUPA-73 RUPA-133 RUPA-126 RUPA-38
j
kon (M-1 s-1)
koff (s-1)
1 nM
10 nM
100 nM
1 μM
10 μM

Fig S1. Single B cell screening assays, domain-specificity of identified mAbs, and affinities of mAbs for Pfs230-C1. Related to **Fig 1** and **Fig 2**. (**a, c**) Representations of the multiplexed bead-based and soluble antigen assays using during primary screening. (**b, d**) Representative microscopic screening images of antibodies assessed for specificity to Pfs230 C1 in each assay. Enzyme-linked immunosorbent assays were performed with Pf230-C1 and Pfs230-Pro constructs to determine domain specificity. Previously identified mAbs against Domain I recognized Pfs230-C1 (**e**), but not Pfs230-Pro (**g**). 16 of the 20 identified mAbs bound to Pfs230-C1 (**f**). Three of these antibodies also react with Pfs230-Pro (**h**). All mAbs were tested at 10 µg/mL. Values are means of three technical replicates and error bars show s.e.m. Dashed lines represent the mean signal + three standard deviations of eight non-Pfs230 mAbs and were used as cutoff to determine positivity. (**i**) Affinity and (**j**) binding kinetics were determined using surface plasmon resonance with immobilized antibodies and antigen as analyte. Black bars and dots represent mAbs with high transmission reducing activity. K_D, binding affinity; k_{on}, association constant; k_{off}, dissociation constant.

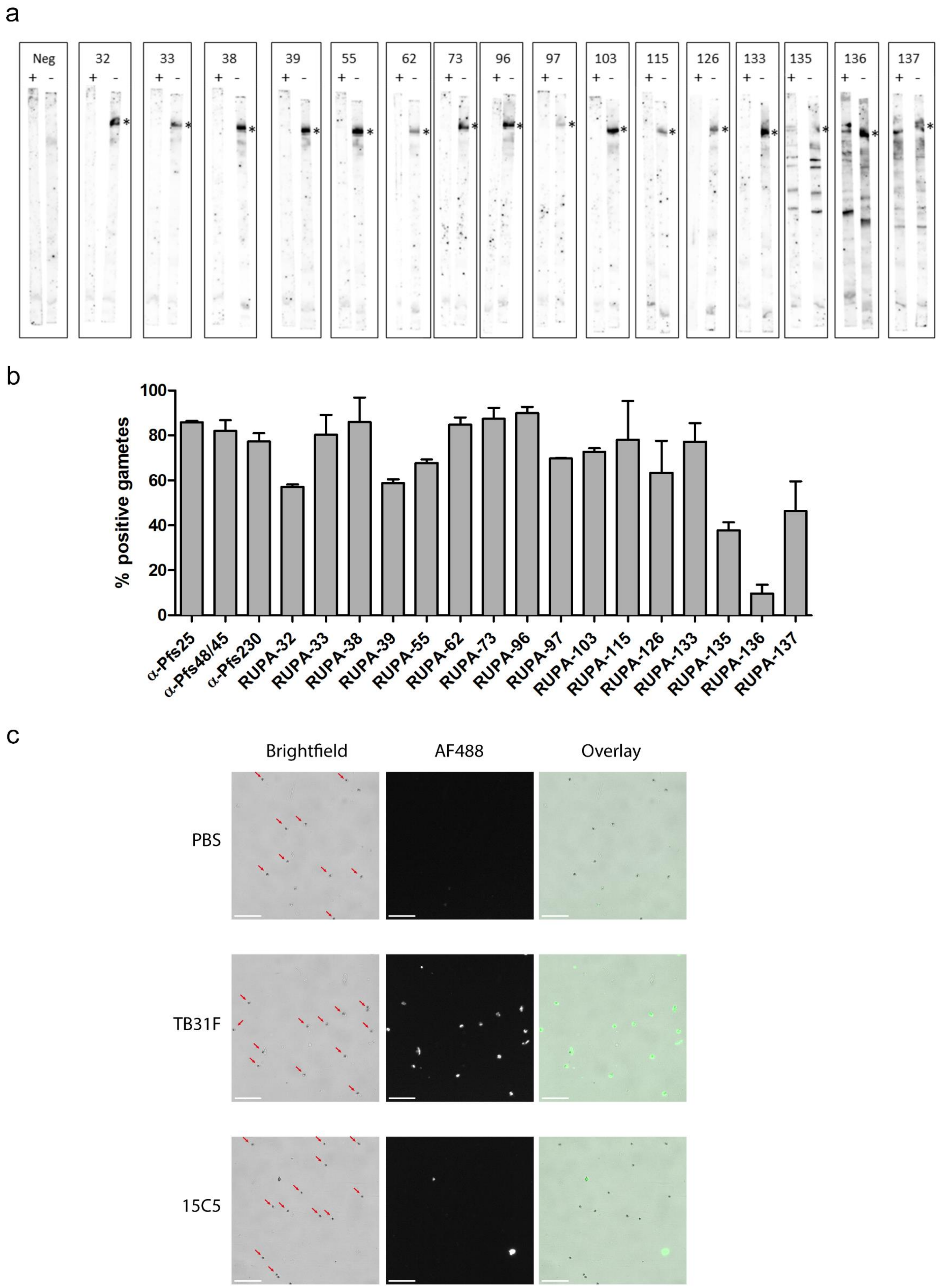
a
Neg
32
33
38
39
55
62
73
96
97
103
115
126
133
135
136
137
b
% positive gametes
0
20
40
60
80
100
α-Pfs25
α-Pfs48/45
α-Pfs230
RUPA-32
RUPA-33
RUPA-38
RUPA-39
RUPA-55
RUPA-62
RUPA-73
RUPA-96
RUPA-97
RUPA-103
RUPA-115
RUPA-126
RUPA-133
RUPA-135
RUPA-136
RUPA-137
c
Brightfield
AF488
Overlay
PBS
TB31F
15C5

Fig S2. Antibody recognition of native Pfs230 by Western blot and surface immunofluorescence assay. Related to **Fig 1**. (**a**) Gametocyte extract was separated on an SDS-PAGE gel under reducing (+) and non-reducing (-) conditions and transferred to a nitrocellulose membrane. Membrane strips were then incubated with different mAbs. Pfs230 band is indicated by asterisks. Neg: non-targeting mAb. (**b**) For SIFAs, all antibodies were tested at 5 µg/mL, except for mAbs against Pfs25 (2544, 1 µg/mL), Pfs48/45 (TB31F, 0.1 µg/mL) and Pfs230 (Hu2A2, 1 µg/mL). Anti-PfCSP mAb 399, which is sporozoite specific and does not recognize female gametes, was used for background correction. Values represent means of two technical replicates after background correction, and error bars show s.d. (**c**) For the micrograph showing that 15C5 does not react with female gametes in surface immunofluorescence assay, TB31F and PBS were included as a positive and negative control respectively. Both TB31F and 15C5 were tested at 3.2 µg/mL. Bound human antibody was detected with anti-human IgG-AF488. Gametes in brightfield channel are indicated with red arrows. Scale bar represents 50 micrometers.

Table S3. X-ray crystallography data collection and refinement statistics.
(Related to **Fig 3** and **Fig 4**.

	Pfs230D1+ and Fab RUPA-32	Pfs230D1+ and Fab RUPA-38	Pfs230D1+ and Fab RUPA-55	Pfs230D1+, Fab RUPA-97, and Fab 15C5	Pfs230D1+ and Fab LMIV230-02
**PDB ID**	7UVH	7UVO	7UVI	7UVQ	7UVS
**Beamline**	APS 23-ID-D	APS 23-ID-B	APS 23-ID-B	APS 23-ID-D	APS 23-ID-B
**Wavelength (Å)**	1.033190	1.033192	1.033167	1.033190	1.033167
**Space group**	C2	C2	I222	$P6_2$	C2
**Cell dimensions**					
**a,b,c (Å)**	154.87, 80.47, 124.96	163.71, 42.85, 95.30	91.02, 100.78, 425.75	155.00, 155.00, 92.90	92.36, 132.47, 125.07
**α, β, γ (º)**	90.0, 111.8, 90.0	90.0, 102.4, 90.0	90.0, 90.0,90.0	90.0, 90.0,120.0	90.0, 102.3, 90.0
**Resolution (Å)**	29.43 - 2.59 (2.68 - 2.59)	29.06 – 2.09 (2.16 – 2.09)	29.67 – 2.92 (3.02 – 2.92)	29.76 – 3.29 (3.41 – 3.29)	29.85 – 2.03 (2.10 – 2.03)
**Total reflections**	540,791 (54,145)	465,926 (34,888)	1,157,261 (118,833)	324,685 (32,881)	651,789 (52,806)
**Unique reflections**	44,578 (4413)	38,739 (3849)	43,033 (4247)	19,432 (1925)	93,278 (8599)
**Multiplicity**	12.1 (12.3)	12.0 (9.1)	26.9 (28.0)	16.7 (17.1)	7.0 (6.1)
**R_{meas}**	0.222 (2.156)	0.219 (1.630)	0.277 (3.627)	0.150 (2.009)	0.124 (1.160)
**R_{pim}**	0.064 (0.615)	0.062 (0.526)	0.054 (0.679)	0.036 (0.481)	0.046 (0.458)
**$\langle I/\sigma I\rangle$**	9.5 (1.6)	7.7 (1.6)	11.5 (1.7)	14.7 (1.7)	11.1 (1.6)
**$CC_{1/2}$**	0.995 (0.696)	0.995 (0.622)	0.998 (0.786)	0.999 (0.648)	0.998 (0.667)
**Completeness (%)**	100 (100)	99.8 (99.4)	99.8 (99.8)	100 (100)	98.6 (90.6)
**Refinement Statistics**					
**R_{work}/R_{free} (%)**	19.6/23.3	21.7/23.5	21.8/23.7	21.5/26.3	17.4/21.5
**Reflections for R_{free}**	1999	1937	2233	987	2019
**Molecules in ASU**	2	1	2	1	2
**Non-H atoms**	9382	4861	9476	8062	10,235
**Macromolecule**	9254	4743	9476	8062	9521
**Water**	88	142	0	0	713
**Heteroatom**	40	6	0	0	1
**RMSD bonds (Å)**	0.011	0.0068	0.0042	0.014	0.0079
**RMSD angles (°)**	1.39	1.01	0.94	1.64	1.05
**Ramachandran statistics**					
**Favored (%)**	97.9	95.9	95.8	96.4	97.7
**Allowed (%)**	2.1	4.1	4.2	3.6	2.3
**Outliers (%)**	0	0	0	0	0
**B-factors ($Å^2$)**					
**Wilson B-value**	57.4	36.7	75.7	121.8	31.6
**Average B-factors**	77.6	64.1	106.5	150.7	49.3
**Average macromolecule**	77.7	64.9	106.5	150.7	49.6
**Average water molecule**	62.0	40.3	-	-	45.5
**Average heteroatom**	80.8	29.1	-	-	52.2

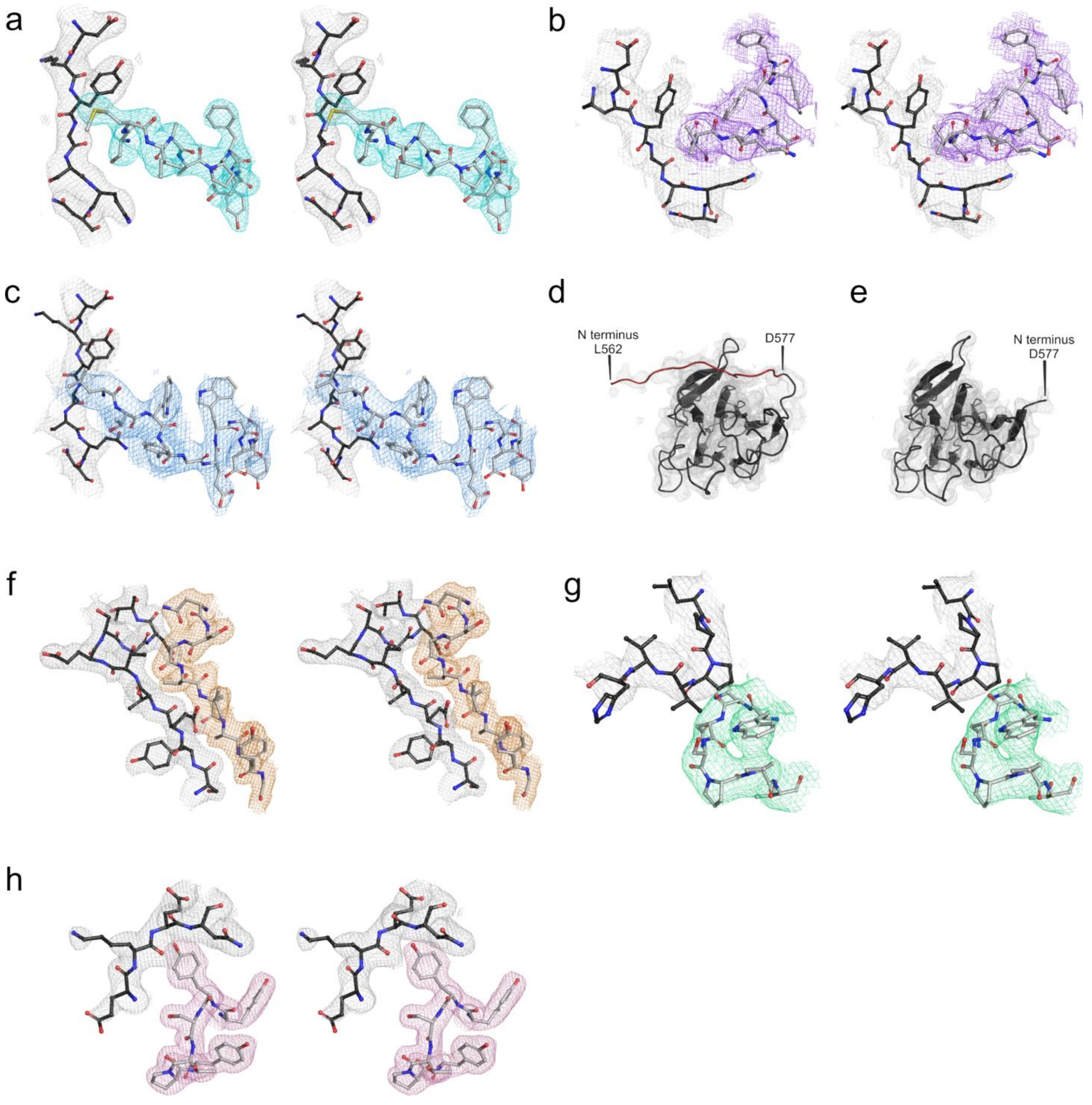

Fig S3. Experimental details of Fab-antigen interactions and Pfs230 D1 structures. Related to **Fig 3** and **Fig 4**. Stereo images of representative composite omit maps depicting electron density contoured at 1.0 sigma around Pfs230 D1+ bound by high potency Fabs (**a**) RUPA -32, (**b**) RUPA-55, and (**c**) RUPA-97, coloured in cyan, purple, and blue, respectively. Composite omit maps depicting electron density contoured at 1.0 sigma around the two biological assemblies of Pfs230 D1+ from the Fab RUPA-55 bound structure observed in (**d**) chain C and (**e**) chain F. The unique residue sequence (amino acids 562 to 576) is highlighted in red. Stereo images of representative composite omit maps depicting electron density contoured at 1.0 sigma around Pfs230 D1+ bound by low potency Fabs, (**f**) RUPA-38, (**g**) 15C5, and (**h**) LMIV230-02, coloured in orange, green, and pink, respectively.

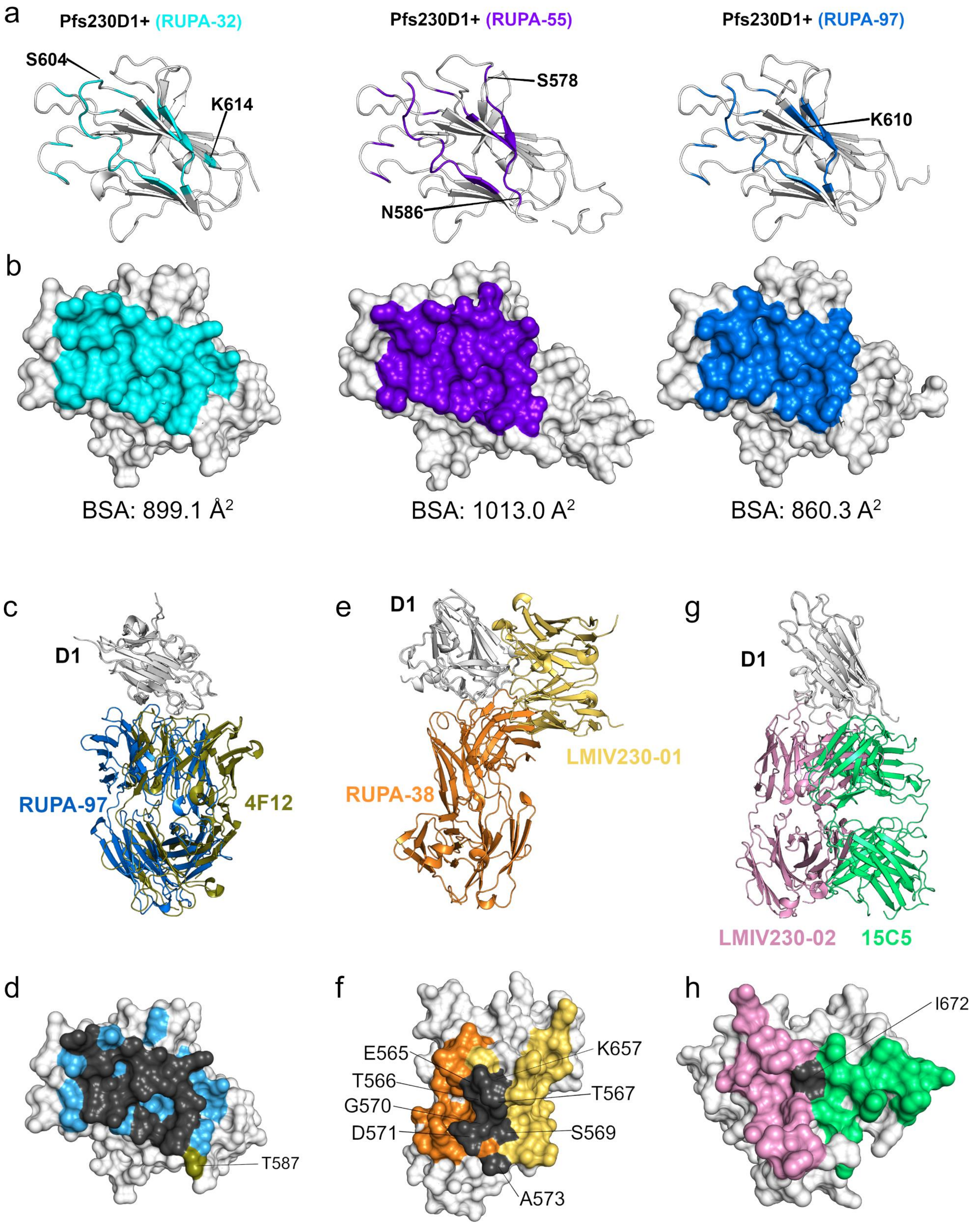

a
Pfs230D1+ (RUPA-32)
Pfs230D1+ (RUPA-55)
Pfs230D1+ (RUPA-97)
S604
K614
S578
N586
K610
b
BSA: 899.1 Å2
BSA: 1013.0 A2
BSA: 860.3 A2
c
D1
RUPA-97
4F12
e
D1
LMIV230-01
RUPA-38
g
D1
LMIV230-02
15C5
d
T587
f
E565
T566
G570
D571
K657
T567
S569
A573
h
I672

Fig S4. Epitopes of high potency transmission-reducing mAbs and other overlapping epitopes on Pfs230 D1. Related to **Fig 3**. The epitopes on Pfs230 D1+ of RUPA-32, -55, and -97 are coloured in cyan, purple, and blue, respectively, shown as (**a**) cartoon and (**b**) surface representations. Unique residues within each epitope are labeled. (**c**) Structural superposition on Pfs230 domain 1 of the Fab RUPA-97 structure with the structure of 4F12 (PDB ID 6OHG). (**d**) Surface representation of Pfs230 D1+ showing the epitopes of RUPA-32, -55, and -97 (light blue) and 4F12 (olive). (**e**) Structural superposition on the Pfs230 domain 1 of the RUPA-38 structure with the structure of scFv LMIV230-01 (PDB ID 7JUM). (**f**) Surface representation of Pfs230 D1+ showing the epitopes of RUPA-38 (orange) and LMIV230-01 (yellow). (**g**) Structural superposition on Pfs230 domain 1 of the Fab LMIV230-02 structure with the structure of Fab 15C5. (**h**) Surface representation of Pfs230 D1+ showing the epitopes of LMIV230-02 (pink) and 15C5 (green). All the overlapping epitopes are coloured in dark gray, and residues are labeled.

Table S4. Co-occurring SNPs present in Pfs230 D1+ amino acids 552 - 731. Related to **Table 1**. No SNPs within epitope bin I (A583T, T602K, E612K, V632A, K716N, and N719S) were found to co-occur with each other, while E612K, A583, and K716 (indicated in bold) co-occurred with SNPs found on other regions of D1+. Since G605S occurs with an allele frequency of 94.4%, the 5.6% of G605 alleles are reported herein.

Co-occurring SNPs	Freq (%)
K661N + D713Y	1.912
T652R + K661N	1.814
G605 + K661N	0.998
T656N + K661N	0.689
E655V + K661N	0.323
K661N + A699T	0.056
K661N + V687I	0.056
G605 + D713Y	0.042
A699T + D713Y	0.014
D561N + K661N	0.014
D637N + K661N	0.014
**E612K** + K661N	0.014
E654K + K661N	0.014
G605 + N715K	0.014
K644Q + K661N	0.014
K661N + H697Q	0.014
K661N + N715K	0.014
K661N + T675K	0.014
K661N + V701M	0.014
K661N + Y726H	0.014
T652R + D713Y	0.014
T652R + K665Q	0.014
V574I + K661N	0.014
T652R + K661N + D713Y	0.351
G605 + T652R + K661N	0.267
E655V + K661N + D713Y	0.070
T652R + T656N + K661N	0.070
K661N + A699T + D713Y	0.042
T652R + E655V + K661N	0.028
T652R + K661N + A699T	0.028
**A583T** + G605 + K661N	0.014
E655V + K661N + A699T	0.014
G605 + K661N + A699T	0.014
G605 + K661N + D713Y	0.014
K661N + D713Y + **K716N**	0.014
K661N + T675K + D713Y	0.014
T656N + K661N + A699T	0.014
T656N + K661N + D713Y	0.014
T652R + K661T + K661N + D713Y	0.042
G605 + E655V + K661N + D713Y	0.014
G605 + T652R + K661N + D713Y	0.014
G605 + T652R + T656N + K661N	0.014
T652R + T656N + K661N + D713Y	0.014
T656N + K661N + A699T + D713Y	0.014

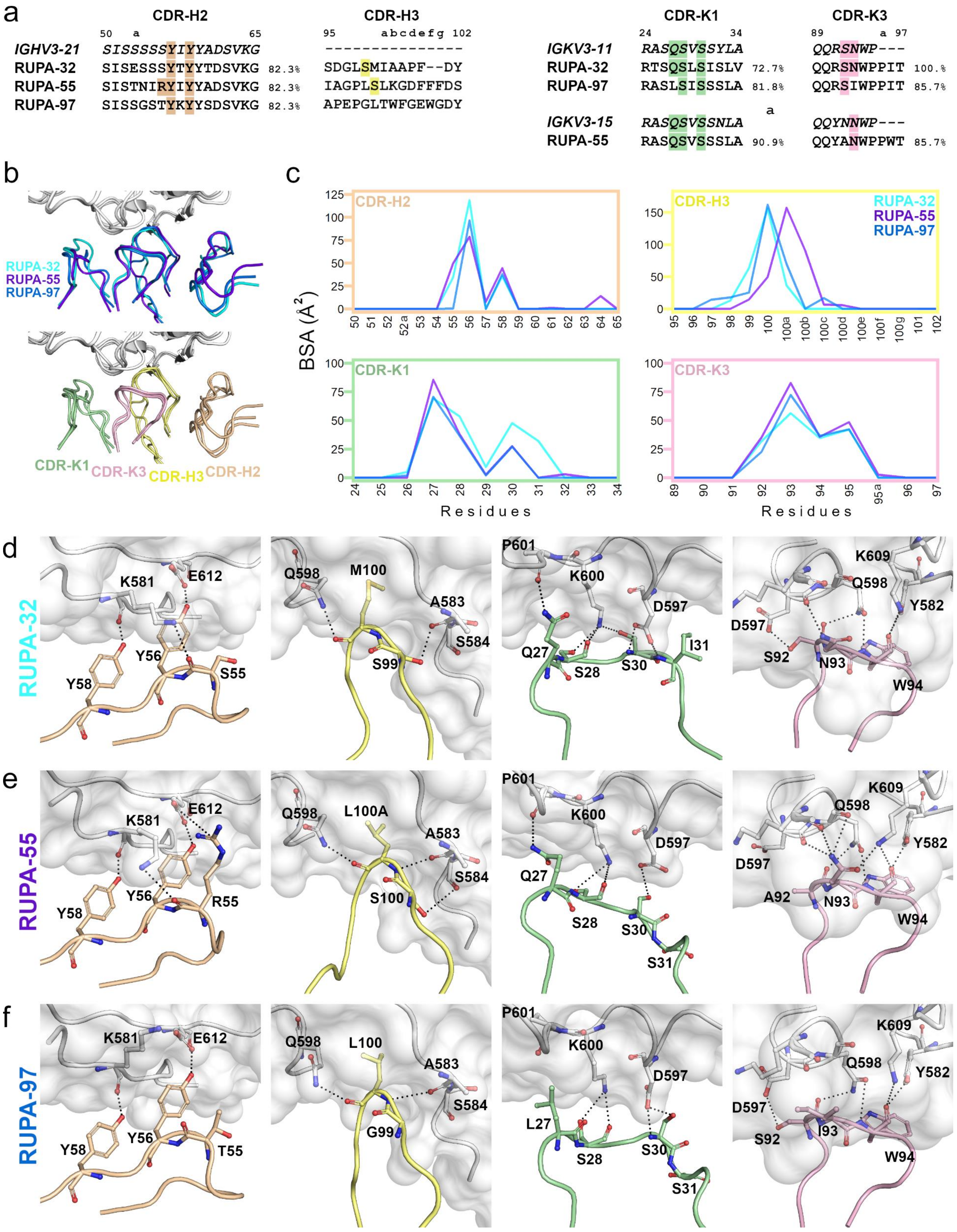
a
CDR-H2
CDR-H3
CDR-K1
CDR-K3
50 a 65
95 abcdefg 102
24 34
89 a 97
IGHV3-21 SISSSSSYIYYADSVKG
RUPA-32 SISESSSYTYYTDSVKG 82.3%
RUPA-55 SISTNIRYIYYADSVKG 82.3%
RUPA-97 SISSGSTYKYYSDSVKG 82.3%

SDGLSMIAAPF--DY
IAGPLSLKGDFFFDS
APEPGLTWFGEWGDY
IGKV3-11 RASQSVSSYLA
RUPA-32 RTSQSLSISLV 72.7%
RUPA-97 RASLSISSSLA 81.8%
QQRSNWP---
QQRSNWPPIT 100.%
QQRSIWPPIT 85.7%
IGKV3-15 RASQSVSSNLA
RUPA-55 RASQSVSSSLA 90.9%
a
QQYNNWP---
QQYANWPPWT 85.7%
b
RUPA-32
RUPA-55
RUPA-97
CDR-K1
CDR-K3
CDR-H3
CDR-H2
c
BSA (Å²)
Residues
RUPA-32
RUPA-55
RUPA-97
d
e
f
RUPA-32
RUPA-55
RUPA-97
K581
E612
Y56
Y58
S55
R55
T55
Q598
M100
L100A
L100
A583
S584
S99
S100
G99
P601
K600
D597
Q27
L27
S28
S30
I31
S31
K609
Y582
S92
A92
N93
I93
W94

Fig S5. Detailed interactions of high potency Pfs230 D1+ binding mAbs. Related to **Table S3** and **Fig 3**. (**a**) Amino acid sequence alignments of antigen-interacting CDRs with the predicted germline precursor sequences. Residues forming H-bonds are highlighted in color. Kabat numbering is used. (**b**) Superposition of all antigen-interacting CDRs of RUPA-32, 55, and 97 in complex with Pfs230 D1+. (**c**) Plots of buried surface area (BSA) contributions by CDR residue. (**d-f**) Detailed interaction interfaces of CDRs K1, K3, H3, and H2 (coloured green, pink, yellow, and tan, respectively) for RUPA-32, 55, and 97. H-bonding interactions are indicated with dashed lines.

Table S6. Hydrogen-bonding interactions between high TRA mAbs and Pfs230 D1+. Related to **Fig 3**. The asterisk (*) denotes a salt bridge.

**Pfs230 D1+**	**RUPA-32**	**RUPA-55**	**RUPA-97**
K581	S55	R55	
K581	Y58	Y58	Y58
Y582	W94	W94	W94
A583	S99	L100a	L100
S584		S100	
D597	S30	S30	S30
D597	I31		
D597	S92		S92
D597	N93	N93	N93
Q598	S92	A92	S92
Q598		N93	
Q598	W94	W94	W94
Q598	M100		L100
Q598		L100a	
K600	S28	S28	S28
K600	S30		
K600		N93	
P601	Q27	Q27	
T602		E1	
E603		E1	
K609		N93	
K609	W94	W94	W94
E612*		R55*	
E612	Y56	Y56	Y56

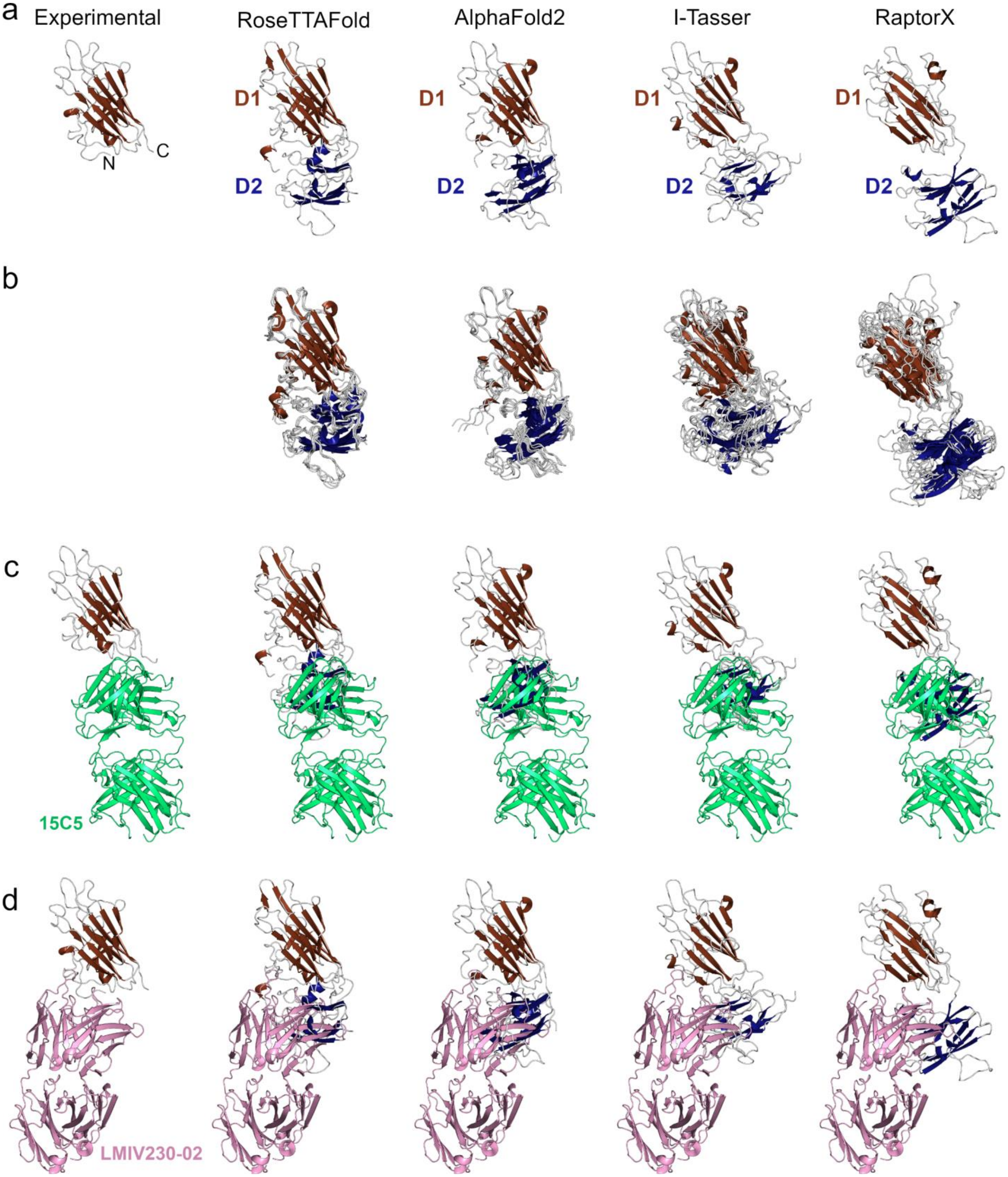

a
Experimental
RoseTTAFold
AlphaFold2
I-Tasser
RaptorX
D1
D2
N
C
b
c
15C5
d
LMIV230-02

Fig S6. Pfs230 domain 2 sterically occludes low-potency epitopes on domain 1. Related to **Fig 4**. (**a**) The crystal structure of Pfs230 D1+ (from the LMIV230-02 complex) contrasted against highest ranked theoretical models of domains 1 and 2 produced by RoseTTAFold[26], AlphaFold2, I-Tasser[27], and RaptorX[28]. (**b**) Superposition at domain 1 of all five output models from each *in silico* modeling approach demonstrates a general convergence for the position of domain 2. Superposition of Fabs (**c**) 15C5 and (**d**) LMIV230-02 onto their respective domain 1 epitopes on the theoretical models of domains 1 and 2 show clashing, independent of which modeling software is used.
